# Morphology-driven gas sensing by fabricated fractals: A review

**DOI:** 10.3762/bjnano.12.88

**Published:** 2021-11-09

**Authors:** Vishal Kamathe, Rupali Nagar

**Affiliations:** 1 Nanomaterials for Energy Applications Lab, Applied Science Department, Symbiosis Institute of Technology, Symbiosis International (Deemed University), Lavale, Pune-412115, Maharashtra, India

**Keywords:** adsorption sites, fabricated fractal, fractal dimension, gas sensor, morphology, pore network, recovery time, response time

## Abstract

Fractals are intriguing structures that repeat themselves at various length scales. Interestingly, fractals can also be fabricated artificially in labs under controlled growth environments and be explored for various applications. Such fractals have a repeating unit that spans in length from nano- to millimeter range. Fractals thus can be regarded as connectors that structurally bridge the gap between the nano- and the macroscopic worlds and have a hybrid structure of pores and repeating units. This article presents a comprehensive review on inorganic fabricated fractals (fab-fracs) synthesized in labs and employed as gas sensors across materials, morphologies, and gas analytes. The focus is to investigate the morphology-driven gas response of these fab-fracs and identify key parameters of fractal geometry in influencing gas response. Fab-fracs with roughened microstructure, pore-network connectivity, and fractal dimension (*D*) less than 2 are projected to be possessing better gas sensing capabilities. Fab-fracs with these salient features will help in designing the commercial gas sensors with better performance.

## Introduction

The industrial sector and its related activities have led to various forms of pollution that compounding up with time. One of the biggest challenges is to control air pollution as it directly affects the respiratory tract and may result in sudden fatalities. History has seen many unfortunate incidents due to the effusion of toxic vapors in the environment [1–4]. Such gas tragedies worldwide in the form of chemical leaks, smoke from fire accidents, and gas leaks from sewage systems, mines and industries, highlight the need of installing efficient gas sensors capable of detecting a range of flammable, poisonous, and harmful gases present in the atmosphere. Gas sensors are alerting systems that are installed in industry setups at both small and large scale, automobile, medical, agriculture, defense, commercial, and residential sectors and thus are versatile regarding their eventual usage [5–7]. The objectives in gas sensing research are usually set to enhance the sensitivity (how the sensor responds to small changes when the gas environment around it changes), selectivity (if a sensor can still respond to a particular gas when many gases present), stability (how the sensor responds in a particular environment with time), the response time (time taken by a sensor to detect no gas to 90% of the gas when exposed to a gas environment), and recovery time (time taken by a sensor to fall to 10% of its baseline resistance value when the gas is removed from the environment). Additionally, low cost, low power consumption, and simple fabrication of gas sensors are desirable factors. Different technologies have been used to detect numerous gases that include semiconductor, catalytic, electrochemical, optical, and acoustic gas sensors [8]. In particular, conductometric semiconductor metal oxide (SMO) sensors are most popular due to their low cost, simplicity, easy fabrication, and wide range of gas detection capabilities [9]. Thin films and nanostructures exhibit better sensing characteristics. Various researchers have reported structures with morphologies such as nanowires (NWs) [10–16], nanorods [17–20], nanosheets [21–23], nanobelts [24–25], and nano/micro-spheres/cubes/polyhedrons [26–29] with enhanced sensitivity as a gas sensor. A rich collection of research articles and review papers on distinct morphological nanostructured gas sensors exist [26,30–31]. Although there are numerous reviews on gas sensing [7,29,32–38], reviews on fabricated fractal (fab-frac)-based gas sensors have not been addressed to the best of our knowledge. In this review, diverse fractal structures used in gas sensing applications are reviewed. The present article first describes what fractals are and what characteristic length scales are associated with fractal growth, followed by material-wise characterization of fab-frac gas sensors discussing their performance and fractal geometries. Some basic definitions are included to aid a non-specialist reader in the field of either fractals or gas sensing to comprehend the discussion. The article finally discusses the role of fractal geometry and identifies key parameters thereof in improving gas detection.

## Review

### What are fractals?

**Figure 1:**
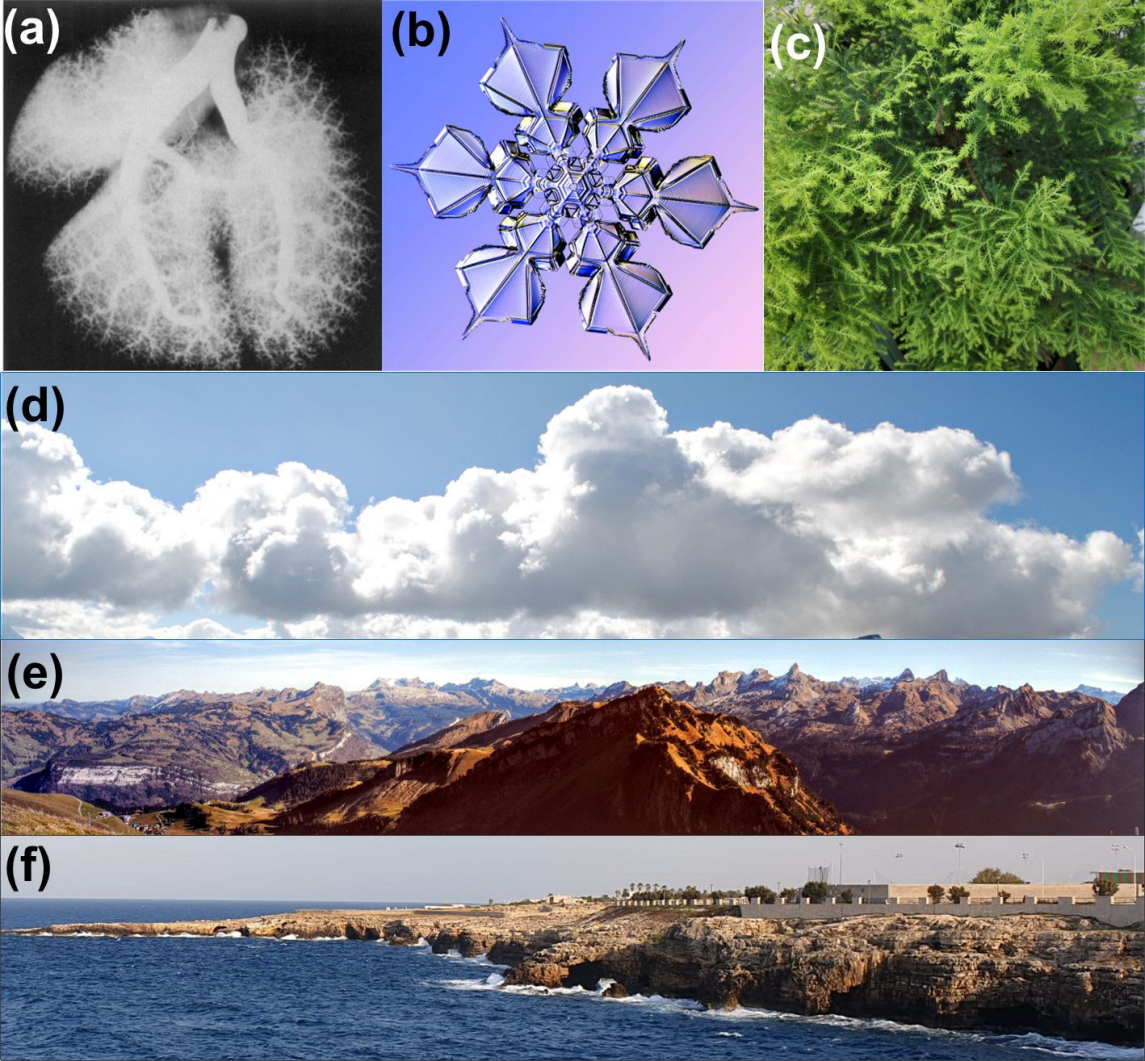
Fractals in nature. Various fractal geometries found in nature: (a) human lung network, (b) snowflakes, (c) leaves, (d) clouds, (e) mountain peaks, and (f) coastline. Image credits: Figure 1a was reproduced from [46], Mary I. Townsley, “Structure and composition of pulmonary arteries, capillaries and veins”, Comprehensive Physiology, with permission from John Wiley & Sons. Copyright © 2012 American Physiological Society. All rights reserved. This content is not subject to CC BY 4.0. Figure 1b was reproduced with permission from [47], SnowCrystals.com by Prof. Kenneth G. Libbrecht. This content is not subject to CC BY 4.0.

B. Mandelbrot, in 1975, coined the term fractal [39–40]. Figure 1 shows various fractal geometries found in nature. Complex patterns seen in human lungs, lines on the surface of human brains, neuron distribution, molecular chains of proteins, and DNA structures with double helix are described by fractal geometries [41]. Ice crystals, lightnings during thunder storms, mountain ranges, and canyons, among many inanimate objects, and some classical patterns such as Koch curves, Cantor sets, and Sierpinski triangles in geometry are also characterized by fractal geometries [41]. These complex, never-ending, replicating patterns across different length scales are termed as fractals. Few researchers have reported fractal growth under lab environment [40,42–45], which are artificially made and are referred to as fab-fracs in this work.

### Fractal length scales and growth models

**Figure 2:**
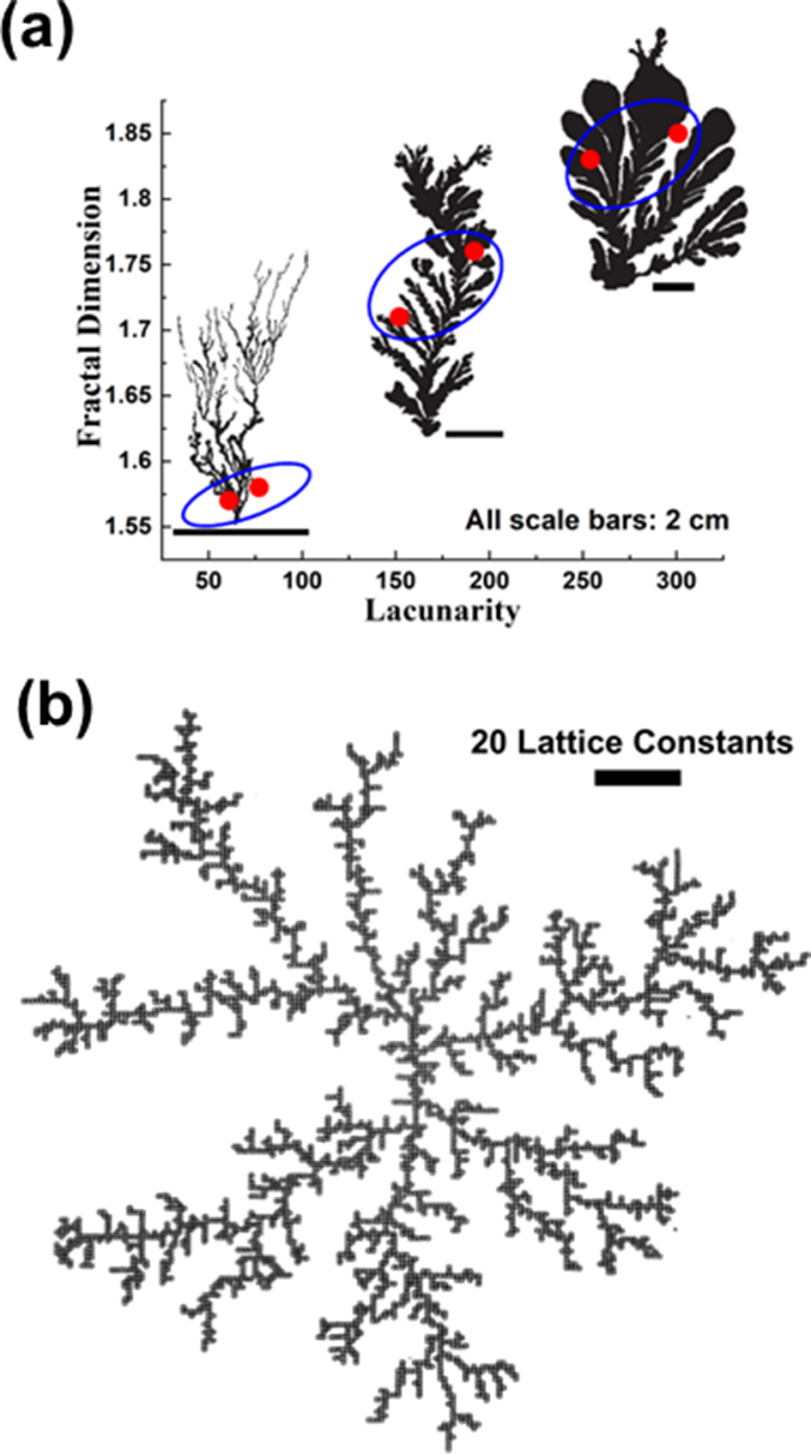
(a) Fractal morphology. Fractal length scales depicting the change in fractal geometry as a function of the fractal dimensions *D* and *L*. Figure 2a was adapted with permission from [48], © 2009 Geological Society of Malaysia. This content is not subject to CC BY 4.0 (b) Diffusion-limited aggregation. Random aggregate of 3600 particles on a square lattice. Figure 2b was reprinted with permission from [52] (T. A. Witten Jr; L. M. Sander, Physical Review Lett., vol. 47, page 1400, 1981). Copyright (1981) by the American Physical Society. This content is not subject to CC BY 4.0.

All fractal geometries have characteristic length scales, namely fractal dimension (*D*), lacunarity (*L*), and connectivity (*Q*), that describe geometric features. Figure 2a shows the examples of different fractal clusters with varying values of *D* and *L* [48]. While *D* measures the complexity of a system, *L* measures the morphological inhomogeneity of fractals. The number *L* also characterizes fractal textures and quantifies the fractal-to-fractal gaps; the higher the lacunarity, the lower is the homogeneity [49]. In fractal studies, surface morphology, geometrical features, and degree of self-organization of materials are determined through these dimensionless numbers [50]. One of the most popular methods employed for determining fractal dimensions is the box-counting method. In this, *N* square grids each of edge length ℓ are placed over an actual optical image or scanning electron micrograph (SEM) with the help of image analysis software. The fractal dimension *D* is then estimated by [43,51]:


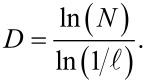


**Figure 3:**
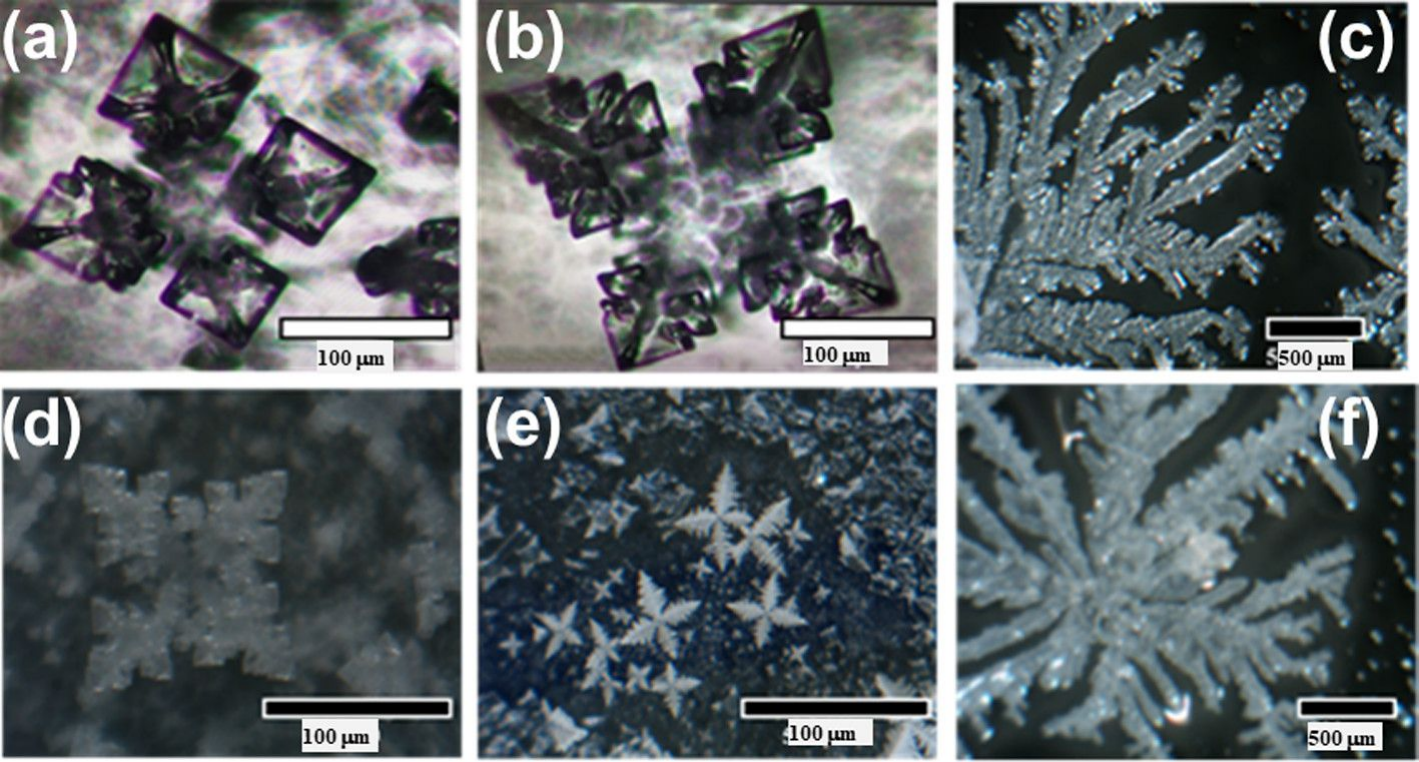
SnO_2_ fab-fracs. Various shapes of large-scale SnO_2_ fab-fracs synthesized under controlled conditions in a lab environment.

This analysis also predicts the power law governing the growth within the fractal space. Growth of fractals in unique geometrical patterns has been predominantly a subject of theoretical treatment. The diffusion-limited aggregation (DLA) model was proposed in 1981 by applying it to the random movement of a metallic ion in a low concentration of electrolyte near a negatively charged electrode [52]. The process resulted in a tree-like scale-invariant structure [52–53]. Figure 2b demonstrates the growth mechanism of a fractal proposed by DLA. Theories of non-equilibrium processes predict pattern formation by considering movement/diffusion of aggregates that ultimately results in the final fractal pattern [53–54]. In the DLA model, one seed particle is placed initially at a location called “origin” of a chosen lattice. Then, another particle is placed far from this origin location. The second particle diffuses via random walk, reaches a site close to the seed particle, and subsequently comes to a stop. In a similar way, other particles are added one by one and allowed to move randomly or guided by diffusion [54]. The added particles eventually reach their final sites and come to rest. The formation of large clusters is thus explained by DLA [52–54]. Like nano/micromaterials, fabricated fractals too, show enhanced sensing abilities due to high porosity (size, number, and pore interconnectivity) and surface area, and high physical connectivity within branched objects [49,55–56]. Figure 3 shows different morphologies of large-scale SnO_2_ fab-fracs grown under controlled lab conditions. The study of the specific surface becomes important for understanding the growth of such structures and investigating the gas sensor characteristics when such structures are used as sensing material [57].

### Proposed growth model for fab-fracs

**Figure 4:**
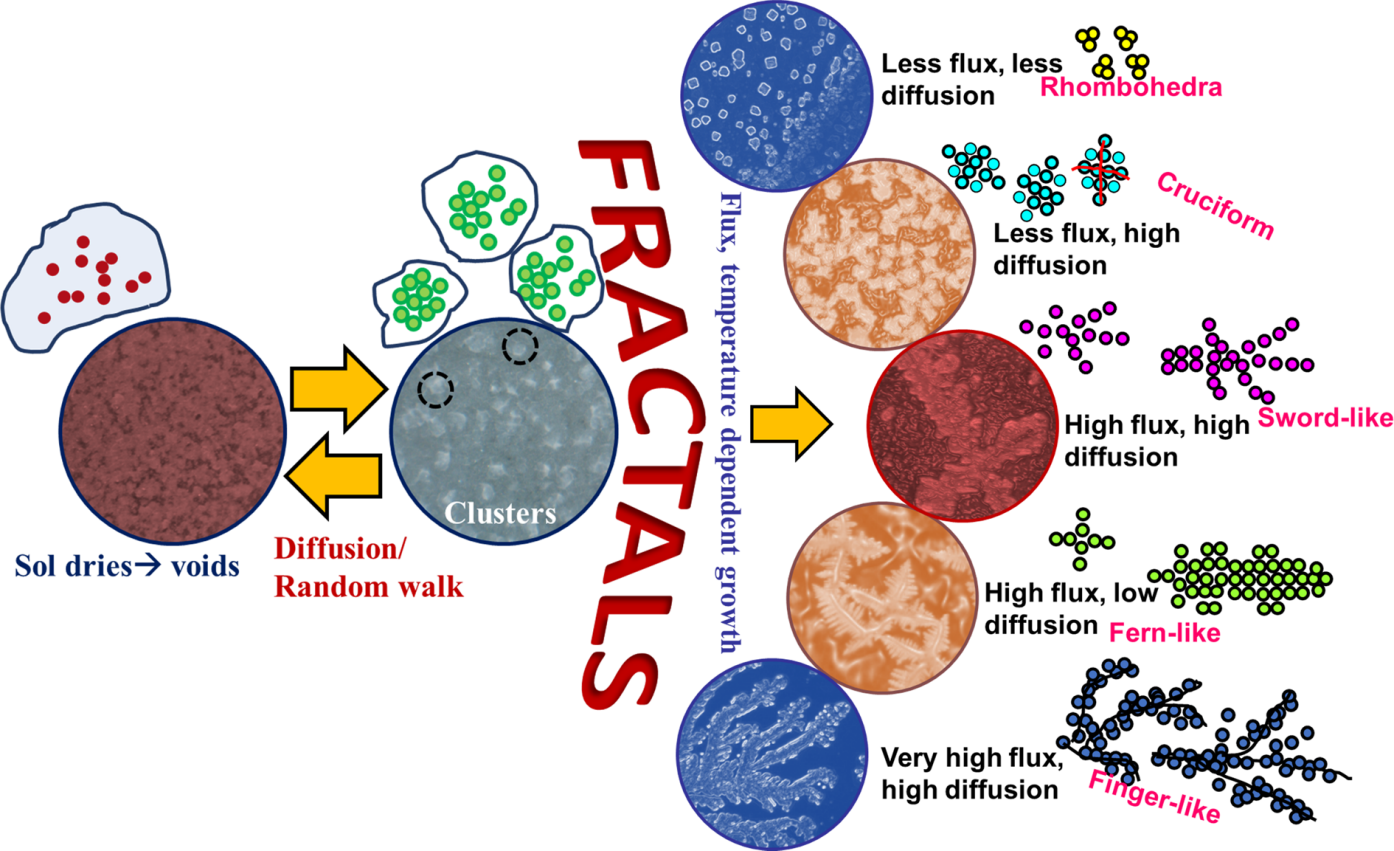
Growth of sol–gel-grown fab-fracs. The schematic depicts the growth mechanism of fab-fracs in different geometries.

This section specifically discusses the various parameters that influence the final shape of a lab-grown fab-frac using the sol–gel technique. Nucleation is a random and probabilistic event that happens on a substrate. As a fluid starts drying, evaporation of the solvent causes voids and helps in generating clusters of the solute on the substrate. For smaller pockets of the fluid, which can be regarded as droplets, variation in surface tension or temperature at different interfaces predominantly causes either a surface tension gradient or a thermal gradient. The diffusivity and dynamic viscosity affect the way in which mass is transported on the substrate. These gradients cause a circulatory flow of fluid, influence the mass transport, and eventually result in differently patterned fractal structures. The effects are, respectively, termed as Gibbs–Marangoni concerning surface tension gradients and Bénard–Marangoni concerning thermal gradients [58–62]. The pattern and shape of the fractals depend on flux, thermal energy, surface energy, and diffusion coefficient of the clusters. The schematic shown in Figure 4 depicts the different stages of fractal formation and conditions that lead to a specific fractal shape. Initially, when the sol starts drying, voids are created due to effusion of gases from the sol. Thereafter, random nucleation and cluster growth takes place. After clusters form on the substrate, further growth into specific fractal shapes depends mainly on availability of sol flux near the growing cluster and the Marangoni effect that includes both thermal energy and diffusion aspects. With limited flux but lower diffusion, rhombohedral fractals are formed that are sparsely spaced on the substrate while with higher diffusion the rhombohedra get very close to assuming a cruciform shape. In the case of high flux and high diffusion, interconnections form on the substrate over a larger scale resulting in longer sword-like fractals. When high flux is available, but diffusion is limited, fractals take the shape of fern-like dendrites. Lastly, with very high flux and high diffusion rates macroscale fractals are obtained. These phenomena are explained in Figure 4 and corresponding optical images of fab-fracs grown in the lab are shown.

### Fractal structures as gas sensors

The complex fractal geometries offer many reasons to be tested for sensing applications. This section reviews the formation of fab-fracs, sorted by the material of the structures, and their performance in gas sensing applications by comparing the fractal dimension, *D*. Wherever the fractal dimension has not been reported, these were estimated using image analysis software. Nano/microscale spheres, cubes, or polyhedra have been applied also as gas sensors. However, these geometries are not discussed in detail in this article due to absence of repeated growth units at different length scales. The interested readers are directed to some excellent articles on hierarchical gas sensors that address such geometries [8,30,33,37].

#### Tin oxide-based fractals

**Figure 5:**
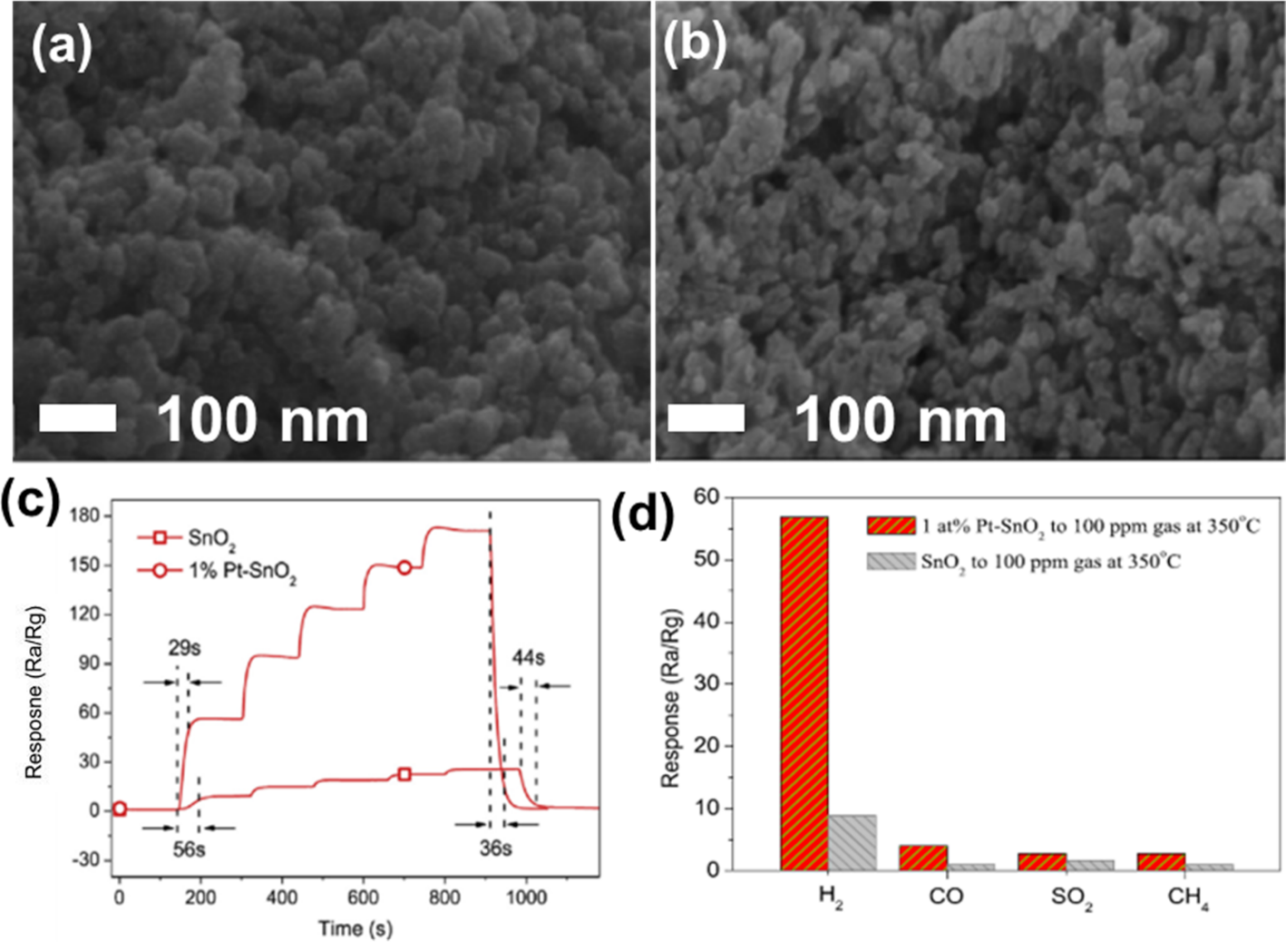
SnO_2_ fractals for H_2_ sensing. SEM images of samples calcined at 550 °C displaying a microstructure of (a) SnO_2_ and (b) 1 atom % Pt-SnO_2_. (c) Response curve of 1 atom % Pt-SnO_2_ for H_2_ ppm level concentrations at 350 °C. (d) Gas sensor selectivity towards 100 ppm of gases/vapors of H_2_ against CO, SO_2_, and CH_4_ at 350 °C. Figure 5a–d was reprinted from [63], Journal of Alloys and Compounds, vol. 805, by X.-T. Yin; W.-D. Zhou; J. Li; Q. Wang; F.-Y. Wu; D. Dastan; D. Wang; H. Garmestani; X.-M. Wang; Ş. Ţălud, “A highly sensitivity and selectivity Pt-SnO_2_ nanoparticles for sensing applications at extremely low level hydrogen gas detection”, pages no. 229–236, Copyright (2019), with permission from Elsevier. This content is not subject to CC BY 4.0.

Yin et al. reported SnO_2_ nanoparticles with and without platinum (Pt) decoration synthesized using a sol–gel hydrothermal technique for gas sensing applications [63]. Figure 5a,b shows SEM images of samples calcined at 550 °C, corresponding to pure SnO_2_ and Pt-SnO_2_, respectively. The box-counting method estimated *D* = 2.43 for pure SnO_2_ and 2.49 for SnO_2_ decorated with 1 wt % Pt, respectively. Figure 5c shows the H_2_ sensing response curve of 1 atom % Pt-SnO_2_. In comparison to pure SnO_2_ and different amounts of Pt decoration (viz. 0.2, 0.5, 1, and 2 atom %), the 1 atom % Pt-decorated SnO_2_ (Pt-SnO_2_) sample exhibited higher sensitivity, a faster response of 29 s at 350 °C, and was able to sense 0.08 ppm H_2_. The sensor response was highest for H_2_ as compared to CO, CH_4_, NO_2_ and SO_2_ gases at 100 ppm gas at 350 degree centigrade. Though the morphology of the fractals did not change appreciably, Pt doping led to faster response and recovery times. This could be due to the excellent interaction of Pt with hydrogen via the established spillover effect that catalyzes hydrogen adsorption [64–65]. Figure 5d depicts the response of fab-fracs with *D* = 2.43 and 2.49 for different gases. Pt decoration can be seen to improve the sensing performance for all analyte vapors, and this can be attributed to the higher catalytic activity due to Pt 5d electrons and the fractal geometry.

**Figure 6:**
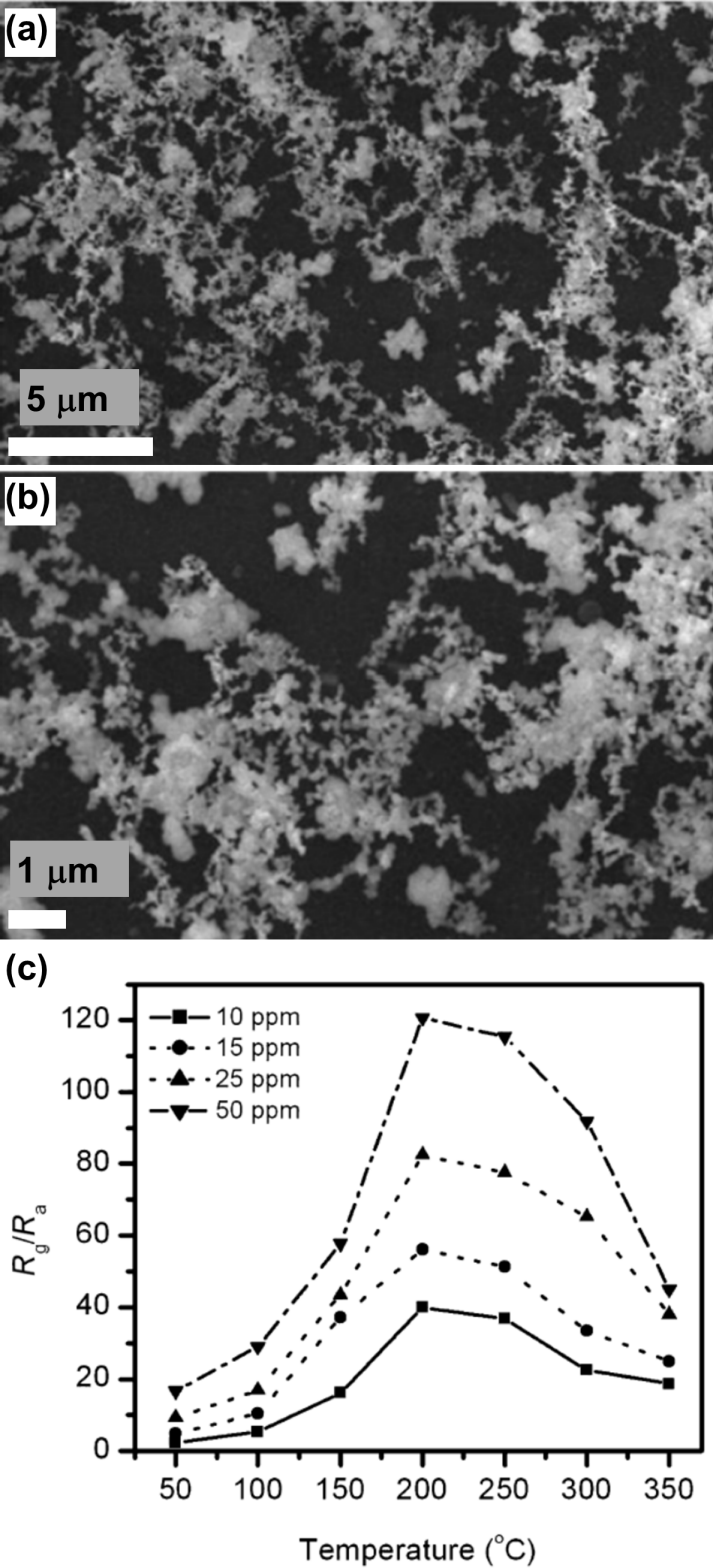
SnO_2_ dendritic nanowires. SEM images of SnO_2_ DNWs at low (a) and high (b) magnifications, respectively. (c) The sensitivity versus temperature curve for 10–50 ppm NO_2_. Figure 6a–c was reprinted from [66], Journal of Alloys and Compounds, vol. 510, by S.H. Mohamed, “SnO_2_ dendrites–nanowires for optoelectronic and gas sensing applications”, pages no. 119–124, Copyright (2012), with permission from Elsevier. This content is not subject to CC BY 4.0.

Plugotarenko et al. employed sol–gel method to prepare SiO_2_·SnO*_x_*·CuO*_y_* nanofilms from a tetraethoxysilane (TEOS) alcohol solution modified by metal salts and applied the samples for NO_2_ sensing [50]. The SiO_2_·SnO*_x_*·CuO*_y_* films annealed at 500 °C exhibited a sample surface consisting of crater-like pits. The self-organization of structures was attributed to tin chloride, which led to a larger size of the pits, while copper oxide led to the formation of hillocks in the film. The *D* values of the samples were in the range of 2.00–2.24. For the Sn/Cu = 6 ratio, the fractal dimension was 2.0, and the sample, exhibiting a combination of hillocks and pores, showed the maximum sensitivity (*S* = 0.29) towards NO_2_. Another study reported dendritic nanowires (DNWs) of SnO_2_ on a gold-coated silicon substrate for NO_2_ sensing [66]. The samples were prepared by evaporation–condensation. Figure 6a and Figure 6b show low- and high-magnification SEM images of SnO_2_ DNWs, respectively. The sensitivity of SnO_2_ DNWs at different temperatures and concentrations of NO_2_ gas is shown in Figure 6c. The sensors exhibited the best performance at 200 °C, at which it was found that the resident oxygen on the sensor surface had minimum influence. Thus, better interaction between NO_2_ and the sensor surface was achieved. The SnO_2_ DNWs were estimated to have a fractal dimension of 1.82.

**Figure 7:**
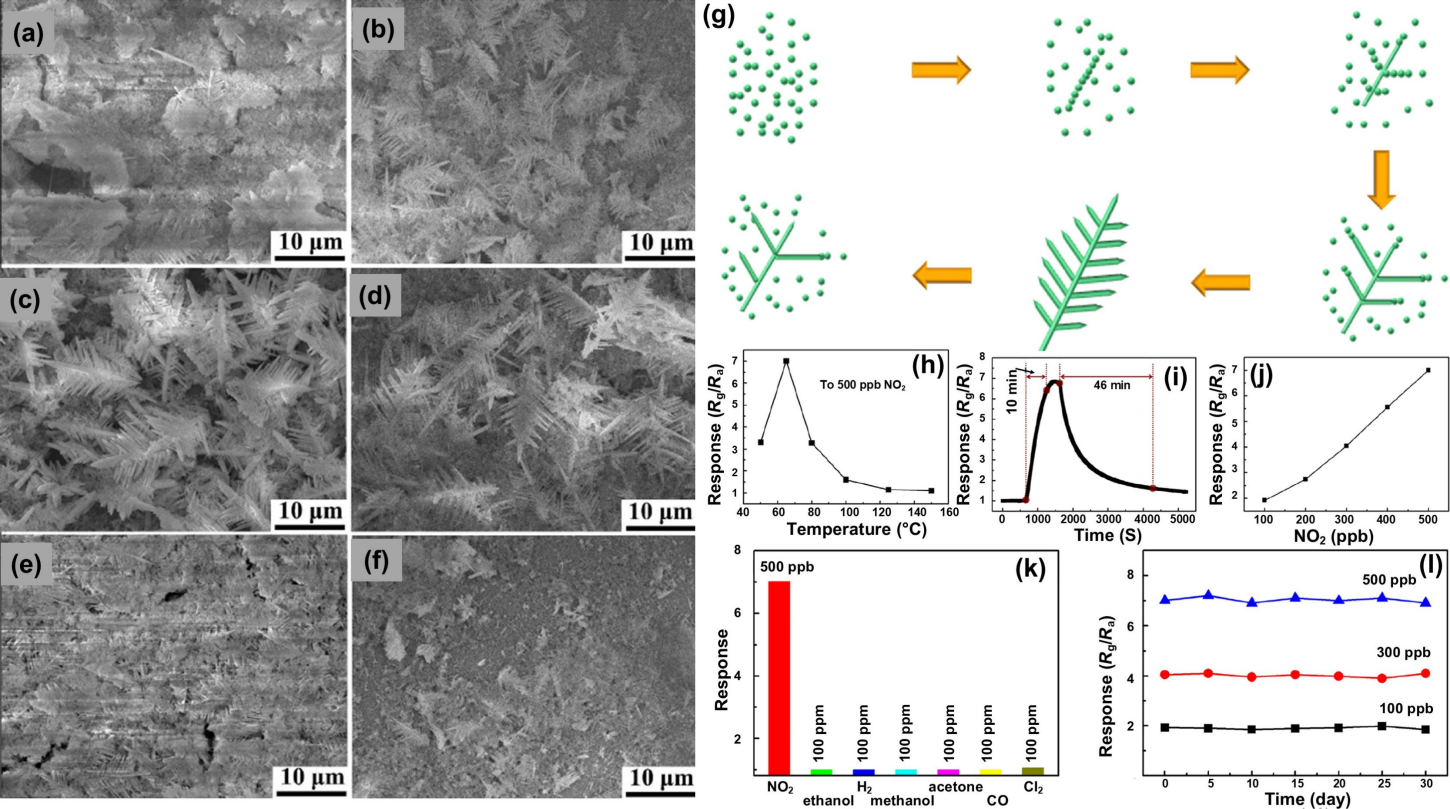
Leaf-like SnO_2_. (a–f) SnO_2_ SEM images of samples obtained after different reaction times, (g) schematic showing the formation of SnO_2_ structures, (h) sensor response at different temperatures, (i) dynamic response curve of the sensor for 500 ppb NO_2_ obtained at 65 °C, (j) sensor response to varying NO_2_ concentrations, (k) response to various gases and (l) stability of the sensor at different concentrations of NO_2_. Figure 7a–l was reprinted from [67], Sensors and Actuators B: Chemical, vol. 255, by Y. Zhang; D. Li; L. Qin; P. Zhao; F. Liu; X. Chuai; P. Sun; X. Liang; Y. Gao; Y. Sun, “Preparation and gas sensing properties of hierarchical leaf-like SnO_2_ materials”, pages no. 2944–2951, Copyright (2018), with permission from Elsevier. This content is not subject to CC BY 4.0.

In 2017, Zang et al. fabricated SnO_2_ leaf-like hierarchical structures by hydrothermal synthesis [67]. Figure 7a–f shows SEM images of SnO_2_ structures after different reaction times. Figure 7g shows a schematic of the formation of hierarchical SnO_2_ structures. Figure 7h–j illustrate the response curves as function of the temperature (Figure 7h), of the time at 65 °C to 500 ppb NO_2_ exposure (Figure 7i), and of the NO_2_ concentration (Figure 7j). The dendritic leaf-like structures provided active sites for the chemical reactions occurring on the surface. Figure 7k and Figure 7l show, respectively, the response of a leaf-like SnO_2_ architecture to different gases and its stability against NO_2_. The fab-frac structure demonstrated enhanced sensing response and better selectivity to NO_2_ at 65 °C. These sensing characteristics were attributed to the dendritic structure promoting diffusion and increasing the availability of adsorption sites. The structures were estimated to have a fractal dimension of 1.78. The results of these three studies show that a lower value of fractal dimension is more effective in sensing NO_2_ gas and lowers the optimum operating temperature.

**Figure 8:**
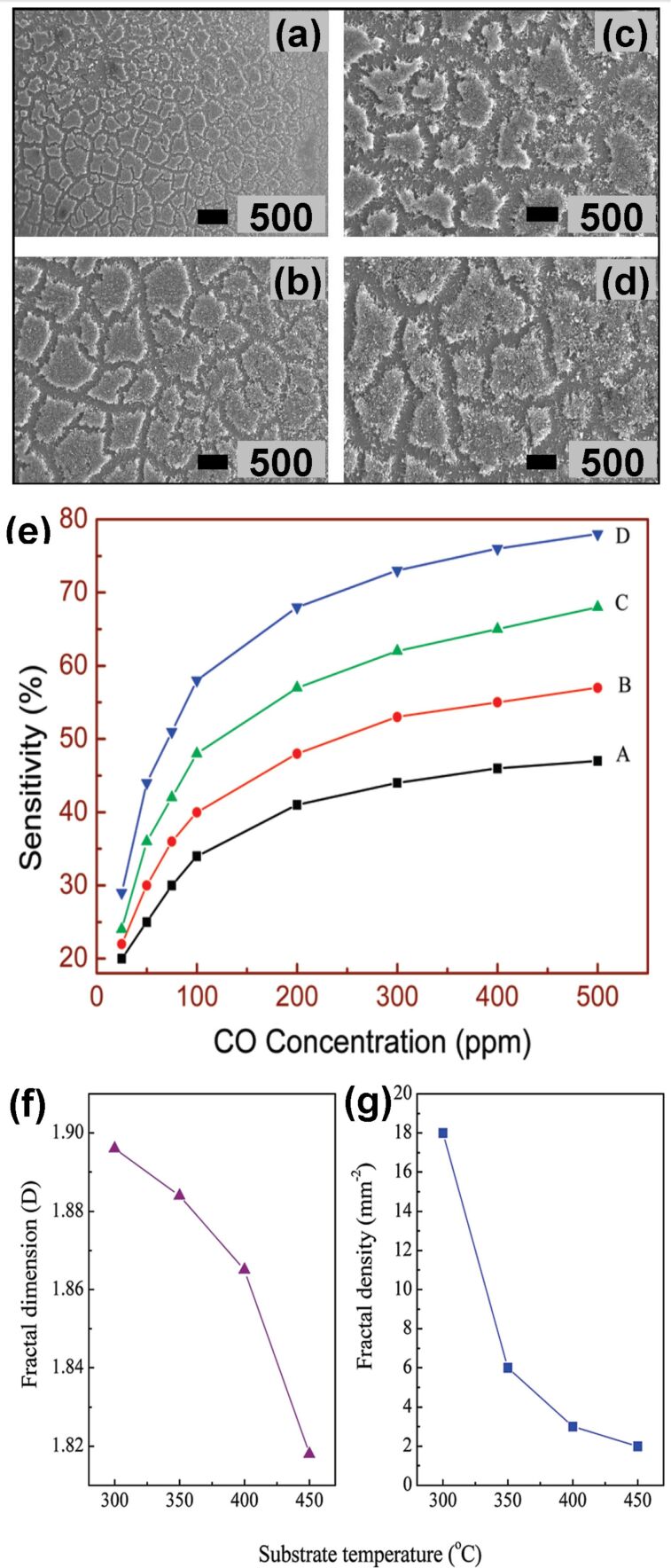
Density and dimension of SnO_2_ fractal films. (a–d) SEM images and (e) CO gas sensing behavior of SnO_2_ thin films prepared on Si(100) substrate at different temperatures (A: 300 °C, B: 350 °C, C: 400 °C, D: 450 °C). (f, g) Influence of the temperature on *D* and fractal density. Figure 8a–g was reproduced with permission from [43], Copyright 2010 American Chemical Society. This content is not subject to CC BY 4.0.

Chen et al. used pulsed laser deposition for growing different SnO_2_ thin films by varying the substrate temperature. The obtained films exhibited fractal features [43]. In another study, Kante et al. prepared SnO_2_ films with fractal morphology by an electrochemical method with a subsequent oxidation process [68]. Both groups tested the films for CO gas sensing at different temperatures. Figure 8a–d shows the SEM images of SnO_2_ thin films on a Si(100) substrate obtained by Chen and co-workers. Figure 8e shows the CO sensing behavior of the fabricated films in the temperature range of 300–450 °C. Figure 8f and Figure 8g show the values of *D* and fractal density as functions of the temperature. The authors concluded that the sensitivity to CO was mainly influenced by channel interconnections, fractal dimension, density, and average size of the fractal clusters. A sensitivity of 0.8 at 450 °C for 500 ppm of CO was achieved. Lower fractal dimension (*D* = 1.818 at 450 °C) and density favored a higher sensitivity towards CO. This could be due to the increased porosity of the structures resulting in more interaction sites at which analyte and sensor can interact. The authors termed the mechanism “random tunneling junction network”. Here, electron transport across the fractal structures is assumed to occur via tunneling. Different fractal dimensions lead to different Schottky barrier heights across the film surface with few locations having a small barrier height depending upon fractal dimension and geometry. Such locations serve as sites with improved sensitivity and respond to the gas faster than other locations that have higher Schottky barrier heights. The gas sensing measurements performed by Kante et al. were in the temperature range of 200–300 °C. For CO, the response was observed to be about 2.5 at 250 °C with a response time of 70 s and a recovery time of 30 s. When exposed to ethanol vapor, the resulting film exhibited a higher sensitivity (400% at 227 °C) towards ethanol with a response time of 140 s. The fractal dimensions of the investigated samples ranged from 1.4 to 1.6. The authors did not observe any dependence on *D*, but a dependence on grain size was reported. These results indicate that a fractal geometry alone is not sufficient to gain better gas sensitivity. Gracheva and co-workers prepared gas-sensitive fractal structures based on SnO_2_ and silicon dioxide (SiO_2_) by a sol–gel technique [57,69–70]. The evolution of fractal aggregates of tin and silicon dioxides resulted in the formation of spherical, labyrinth, and percolation network structures. The spherical and labyrinth structures exhibited low sensitivity to ethanol and acetone vapors, while a sensitivity greater than 20 it was observed in case of percolation network nanostructures. Thus, the network and pore connectivity of fractal nanostructures becomes crucial for a better gas sensing response. Similar observations on the importance of network interconnectivity have been made by Chen et al. as discussed above.

**Figure 9:**
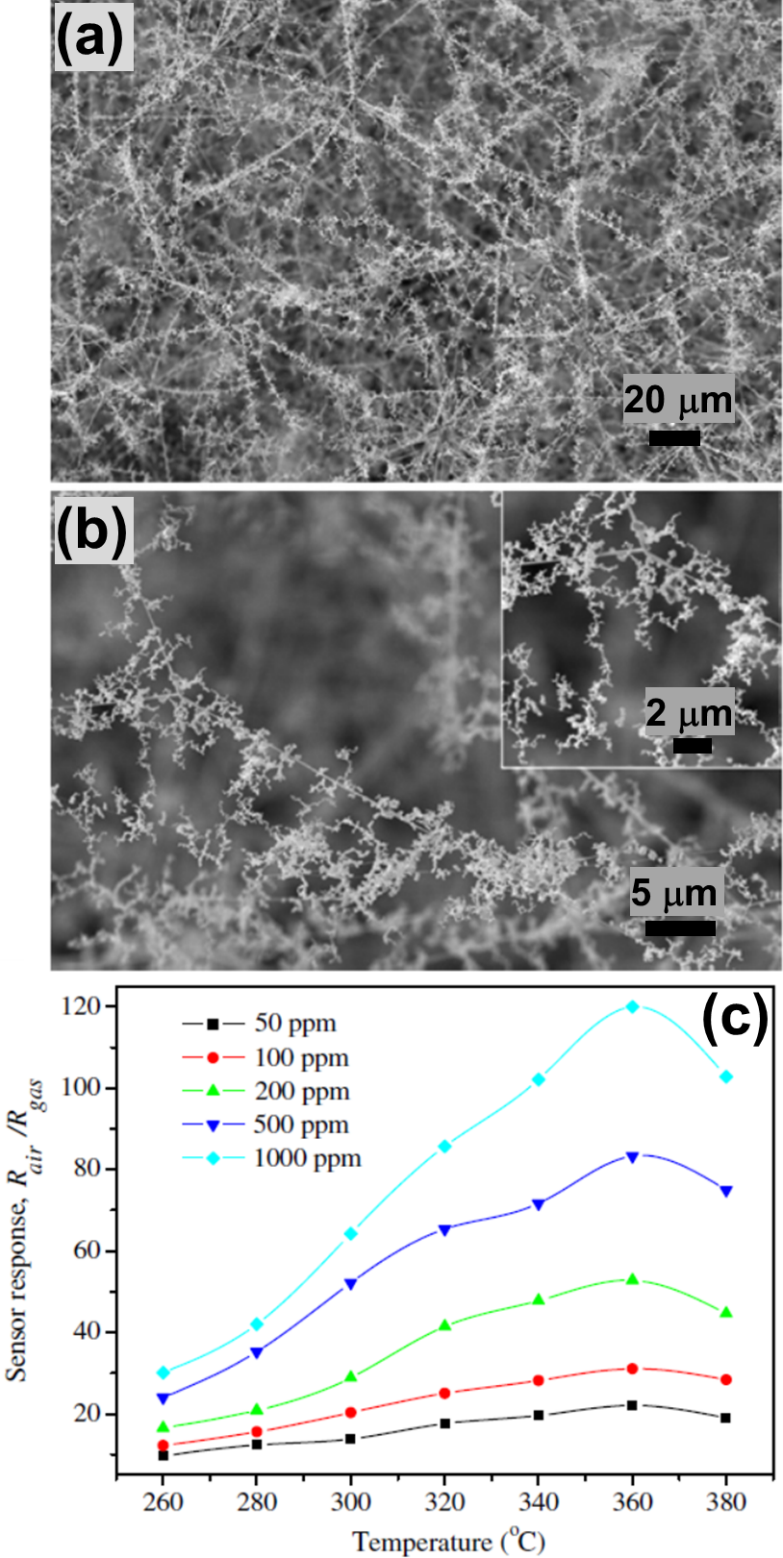
SnO_2_ fractal ethanol sensors. Typical SEM images with (a) low and (b) high magnification of the grown SnO_2_ nanostructures (insets show linkages of nanodendrites). (c) The sensor response as function of the temperature with different C_2_H_5_OH concentrations. Figure 9a–c was reprinted from [71], Current Applied Physics, vol. 11, by S. Phadungdhitidhada; S. Thanasanvorakun; P. Mangkorntong; S. Choopun; N. Mangkorntong; D. Wongratanaphisan, “SnO_2_ nanowires mixed nanodendrites for high ethanol sensor response”, pages no. 1368–1373, Copyright (2011), with permission from Elsevier. This content is not subject to CC BY 4.0.

In 2011, Phadungdhitidhada et al. prepared SnO_2_ NWs of different diameters (from 50–150 nm) with and without nanodendrites (NDs), with lengths extending to a few tens of micrometers and NDs of 100–300 nm in diameter by closed crucible carbothermal reduction of SnO_2_ [71]. Figure 9a and Figure 9b show SEM micrographs of the synthesized SnO_2_ structures at lower and higher magnification, respectively. Figure 9c illustrates the sensor response at different temperatures for different concentrations of ethanol. The SnO_2_ with NDs showed enhanced ethanol sensing in comparison to SnO_2_ NWs without NDs, which was attributed to a higher surface-to-volume ratio, more grain boundaries, and the presence of junction barriers at the ND–NW interfaces. The estimated *D* for the SnO_2_ nanodendrites was 1.88.

**Figure 10:**
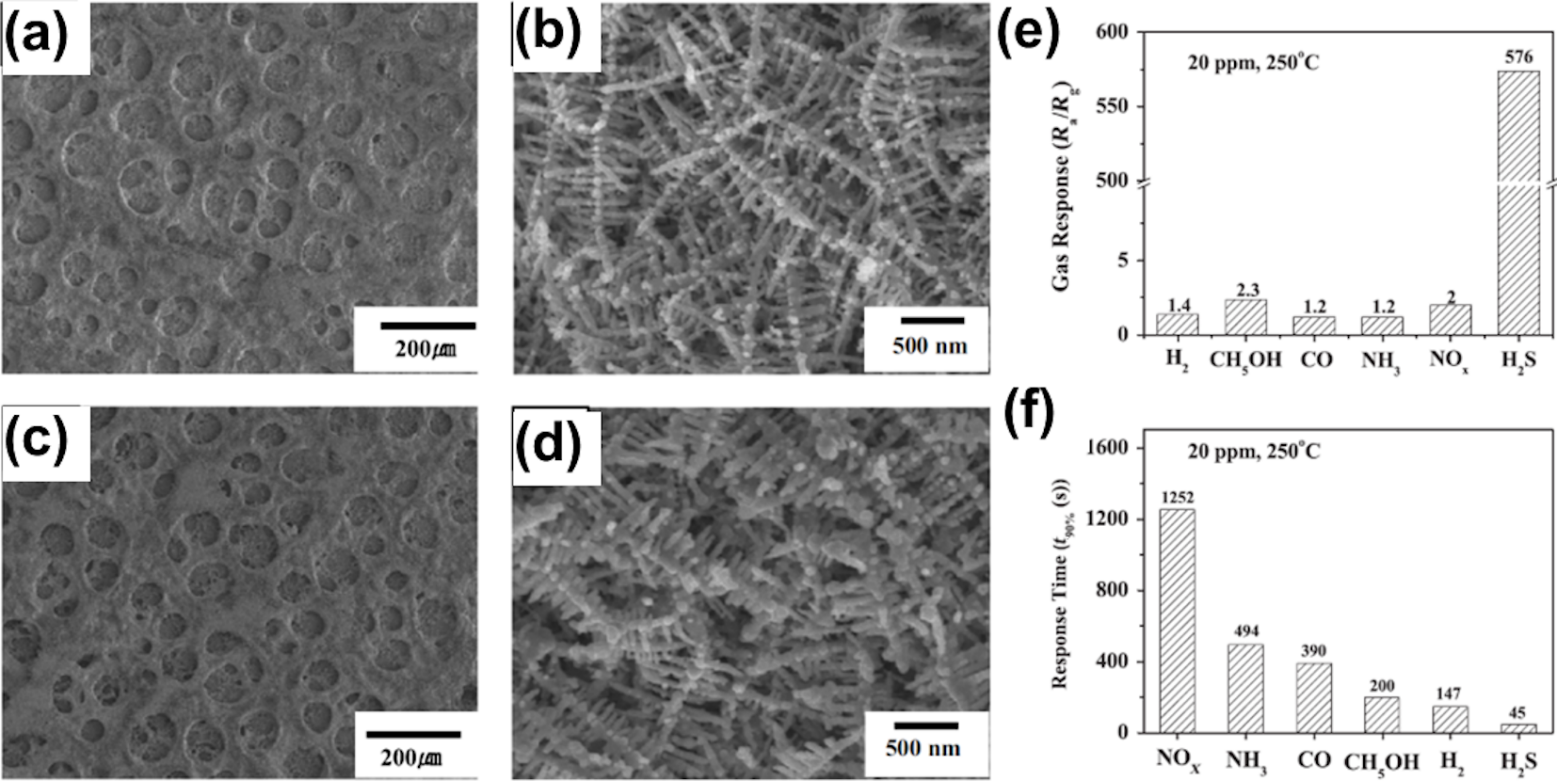
SnO_2_/CuO nanoscale hybrid dendrites. SEM images of (a) Sn–Cu porous foams, (b) the 3D interlock network structure of the as-deposited sample, (c) oxide foam annealed at 700 °C, and (d) the structure of dendrites formed in the pore wall at 700 °C. (e) Gas response and (f) response time of the SnO_2_/CuO nanoscale hybrid foam sensor to 20 ppm of H_2_, C_2_H_5_OH, CO, NH_3_, NO*_x_*, and H_2_S at 250 °C. Figure 10a–f was reprinted from [72], Materials Letters, vol. 105, by J.-H. Jeun; D.-H. Kim; S.-H. Hong, “SnO_2_/CuO nano-hybrid foams synthesized by electrochemical deposition and their gas sensing properties”, pages no. 58–61, Copyright (2013), with permission from Elsevier. This content is not subject to CC BY 4.0.

3D porous nanoscale hybrid SnO_2_/CuO foam sensors were prepared by Jeun et al. via electrochemical deposition followed by thermal oxidation [72]. These foam sensors were studied for H_2_S gas sensing. Figure 10a and Figure 10b show SEM images of the porous and 3D network structure of as-prepared Sn/Cu foam after electrodeposition. Figure 10c and Figure 10d illustrate the porous foam structure formed at 700 °C by thermal oxidation and the dendritic structures formed in pore wall. The foam sensor was able to detect down to 4 ppm of H_2_S. The highest gas response (*S* = 576) was obtained for 20 ppm of H_2_S at 250 °C. The study shows that the SnO_2_/CuO nanoscale hybrid foam sensor outperforms the porous 3D network structure, mainly due to larger surface area, the formation of p–n junctions, and the sulfurization of CuO on metallic conductors. The foam sensor also showed a response to 20 ppm of hydrogen, carbon monoxide, ammonia, nitrous oxide, and ethanol (Figure 10e,f) at 250 °C. The estimated fractal dimensions were 1.82 for the pore network and 1.72 for the foam sensor.

#### Titanium oxide-based fractals

**Figure 11:**
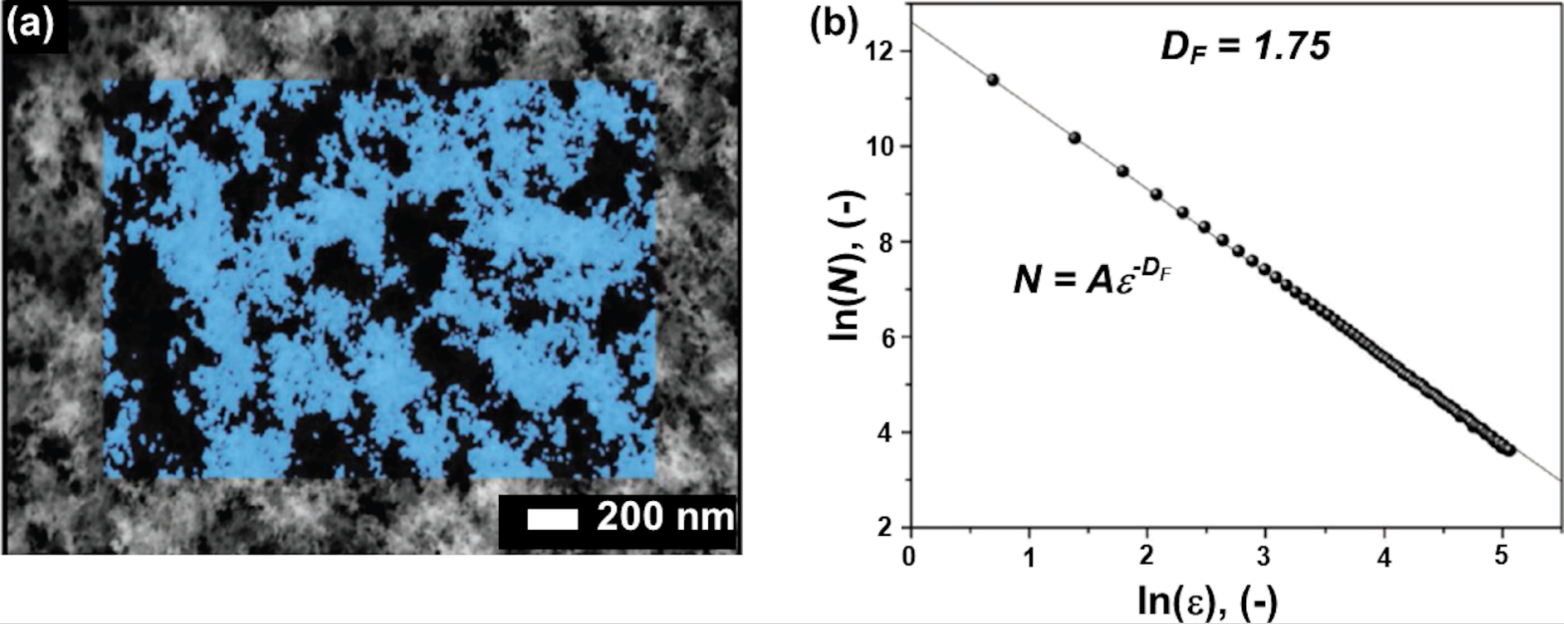
TiO_2_ fractals. (a) Top-view SEM image of TiO_2_ fractals. (b) Double-log plot of foreground pixel number with the scaling factor. The estimated fractal dimension was 1.75. Figure 11a,b was reproduced from [49], Z. Fusco, M. Rahmani, R. Bo, R. Verre, N. Motta, D. Neshev, A. Tricoli, “Nanostructured Dielectric Fractals on Resonant Plasmonic Metasurfaces for Selective and Sensitive Optical Sensing of Volatile Compounds”, Advanced Materials, with permission from John Wiley & Sons. Copyright © 2018 WILEY-VCH Verlag GmbH & Co. KGaA, Weinheim. This content is not subject to CC BY 4.0.

Fusco et al. modified dielectric titanium oxide (TiO_2_) nanoparticles with fractal structure with a plasmonic gold (Au) metasurface for sensing volatile organic compounds (VOCs) [49]. This modification enhanced the plasmonic field and local surface plasmonic resonance (LSPR). The influence of the gold nanodisk diameter and the average thickness of the TiO_2_ fractal on LSPR sensing of VOCs, specifically ethanol, acetone, and toluene, was examined. The LSPR sensor showed a 4–8 times higher sensitivity for detecting gas molecules with the fractal-enhanced dielectric structure. The enhancement in the sensitivity was mainly attributed to the large surface-to-volume ratio of fractal system, which resulted in a higher probability of volatile gases condensing, and the enhancement of the electrodynamic field above the Au surface. These factors were vital only up to certain thickness above which it was believed that the volatile gases penetrate into the fractal volume followed by diffusion to active sensing regions resulting in decreased sensitivity. Figure 11a and Figure 11b show, respectively, a SEM image and the scaling factor with fractal dimension *D* = 1.75 of the TiO_2_ fractals.

**Figure 12:**
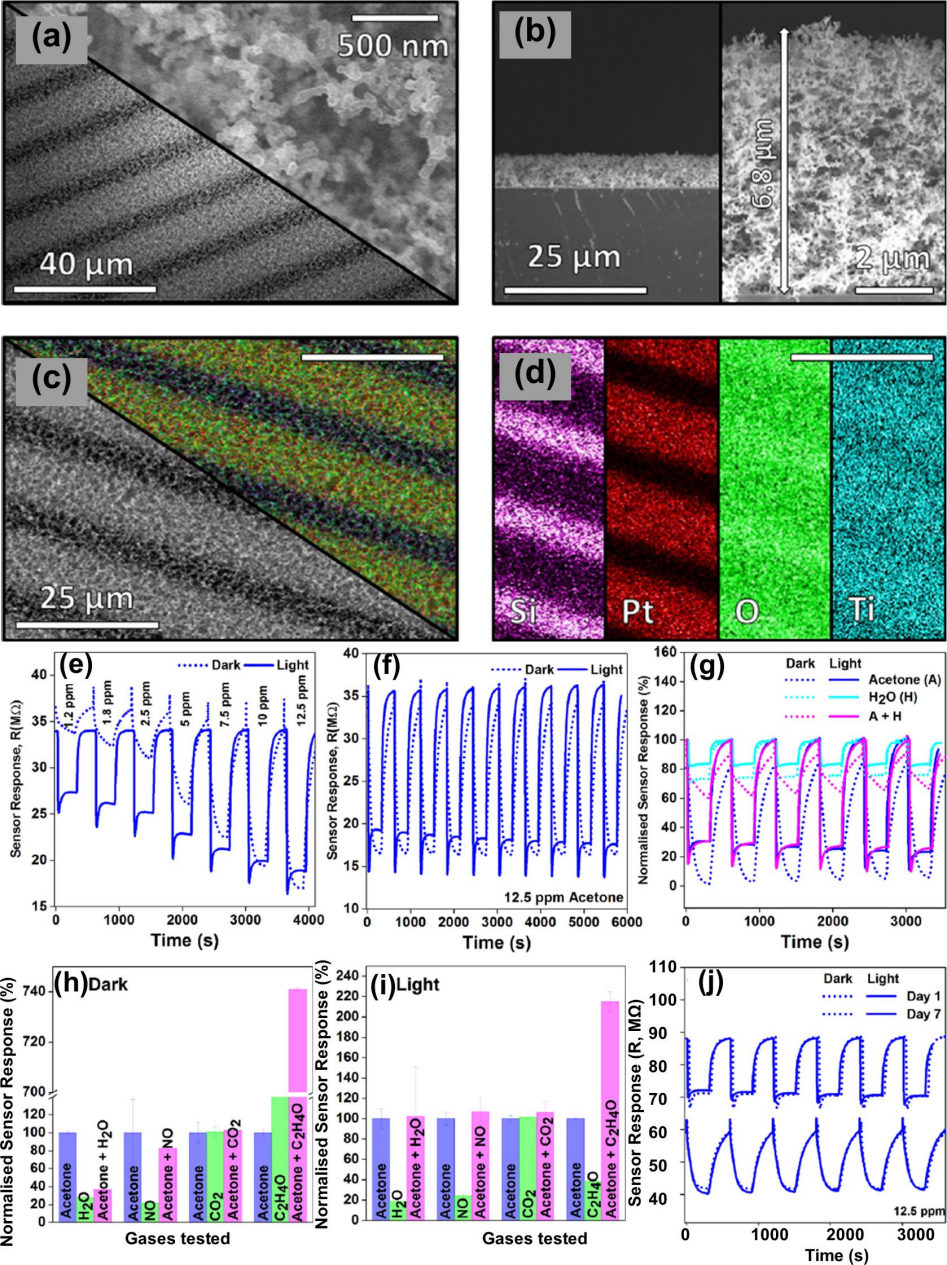
TiO_2_ fractals on candle soot templates. SEM images of (a) top-view morphology and (b) thickness of the specimen. (c) EDS maps of the deposited ST material on the sensor. (d) EDS maps for single elements Si, Pt, O and Ti of soot derived TiO_2_ layers on Pt electrode-based sensor, (e–j) Optical and photo-response results of ultra-porous TiO_2_ films. Figure 12a–j were reprinted from [73], Sensors and Actuators B: Chemical, vol. 275, by Y.M. Sabri; A.E. Kandjani; S.S.A.A.H. Rashid; C.J. Harrison; S.J. Ippolito; S.K. Bhargava, “Soot template TiO_2_ fractals as a photoactive gas sensor for acetone detection”, pages no. 215–222, Copyright (2018), with permission from Elsevier. This content is not subject to CC BY 4.0.

Sabri et al. synthesized a TiO_2_ structure referred to as soot-derived TiO_2_ layers (ST) [73]. These were formed on a Pt electrode resulting in a sensor prototype and were later used for UV-assisted acetone sensing. Figure 12a and Figure 12b show SEM images of top-view morphology and thickness of the sample at low and high magnifications, respectively. Figure 12c shows an energy-dispersive X-ray spectroscopy (EDS) mapping of the sensor, while Figure 12d shows individual EDS maps of Si, Pt, O, and Ti. The fabricated sensor displayed good sensitivity towards acetone under exposure to UV light with a detection limit greater than 97% at 10 ppb. The exceptional sensitivity achieved was attributed to high porosity, network structure, and large surface area of fractal structure. Figure 12e–j shows the optical and photo response of ultra-porous TiO_2_. These structures were estimated to have a fractal dimension of 1.77.

#### Iron oxide-based fractals

**Figure 13:**
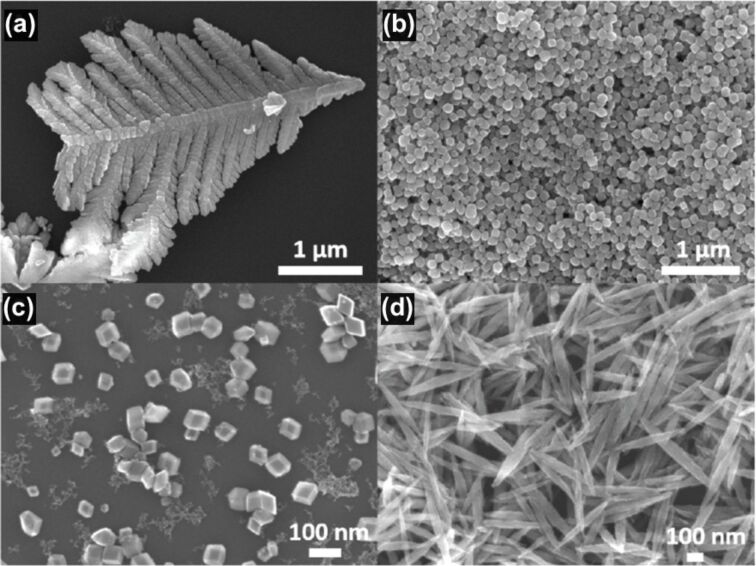
Hematite fractals. SEM images of (a) dendrites, (b) nanocubes, (c) rhombohedra, and (d) spindles of α-Fe_2_O_3_. Figure 13a–d was reprinted from [74], Procedia Engineering, vol. 120, by G. Bailly; J. Rossignol; B. de Fonseca; P. Pribetich; D. Stuerga, “Shape-controlled synthesis of hematite for microwave gas sensing”, pages no. 764–768, Copyright (2015), with permission from Elsevier. This content is not subject to CC BY 4.0.

Bailly et al. fabricated dendrites, cubes, rhombohedra, and spindle-shaped hematite α-Fe_2_O_3_ fractal crystals by a cost-effective and eco-friendly microwave method [74]. Figure 13 shows SEM images of different hematite crystals obtained by varying precursor concentrations and additives. A dendritic particle structure with a middle stem of 3.5 µm and secondary branches of ca. 1 µm to 250 nm, a structure of 700 nm long spindle particles, a rhombohedral structure of 80 nm, and a cubic particle structure of 100 nm were obtained. The estimated fractal dimensions for dendrites, cubes, rhombohedra, and spindles were 1.56, 1.89, 1.49 and 1.81, respectively. The sensing material was deposited on an antenna, and a microwave transduction principle was employed for gas sensing. In these measurements, the interaction of the gas analyte with the sensor was studied at different frequencies and changes in the reflection coefficient and dielectric properties of sensing material were observed. The response of sensor was described by the real and imaginary parts of the reflection coefficient using a specific waveguide. The response was found to be linear for ammonia in the range of 0–500 ppm.

#### Zinc oxide-based fractals

**Figure 14:**
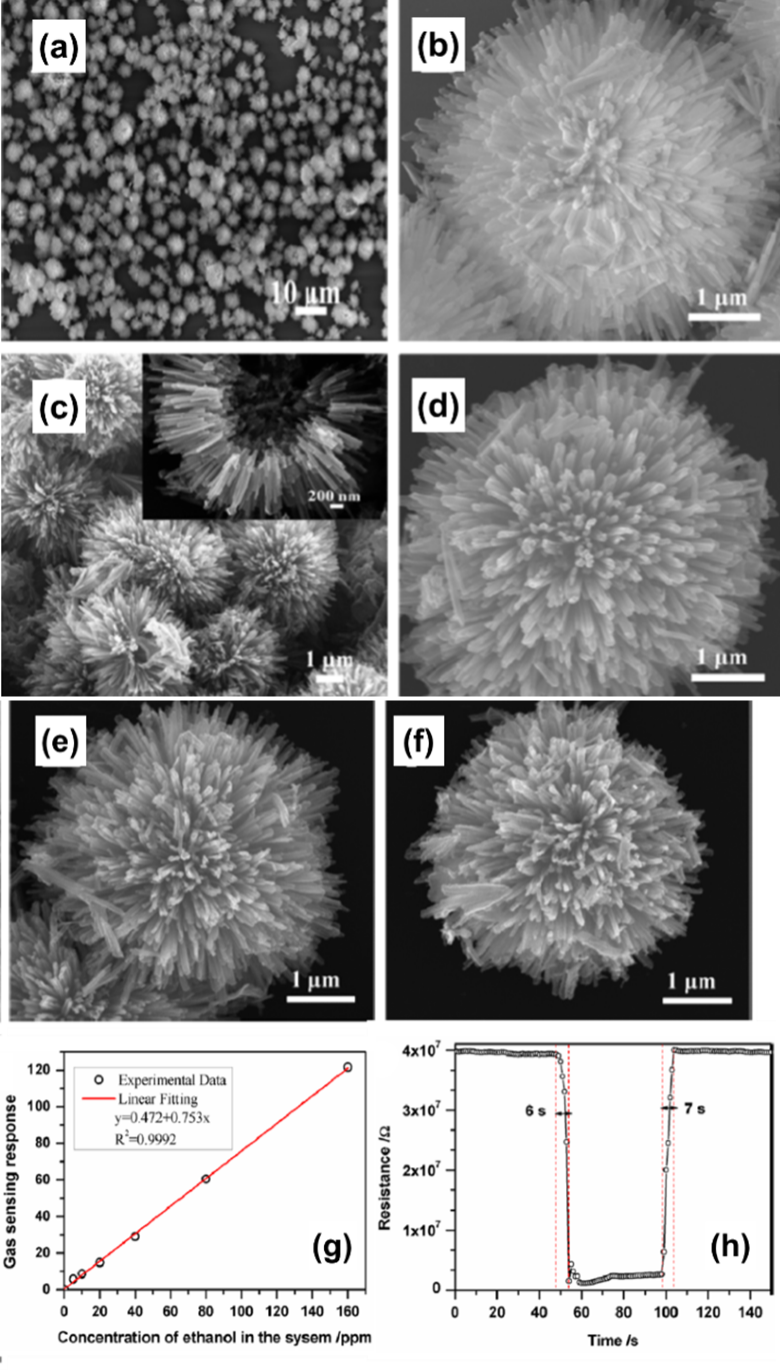
Dandelion-like ZnO fractals. (a–f) SEM images of the samples annealed at different temperatures. The inset in (c) shows the hollow structure of ZnO-350, that is, the sample annealed at 350 °C. (g) Response and corresponding linear fit for ZnO-350 to different concentrations of ethanol at 250 °C. (h) Response to 50 ppm ethanol and recovery time of ZnO-350 at an operating temperature of 250 °C. Figure 14a–h were reproduced with permission from [75], Copyright 2014 American Chemical Society. This content is not subject to CC BY 4.0.

**Figure 15:**
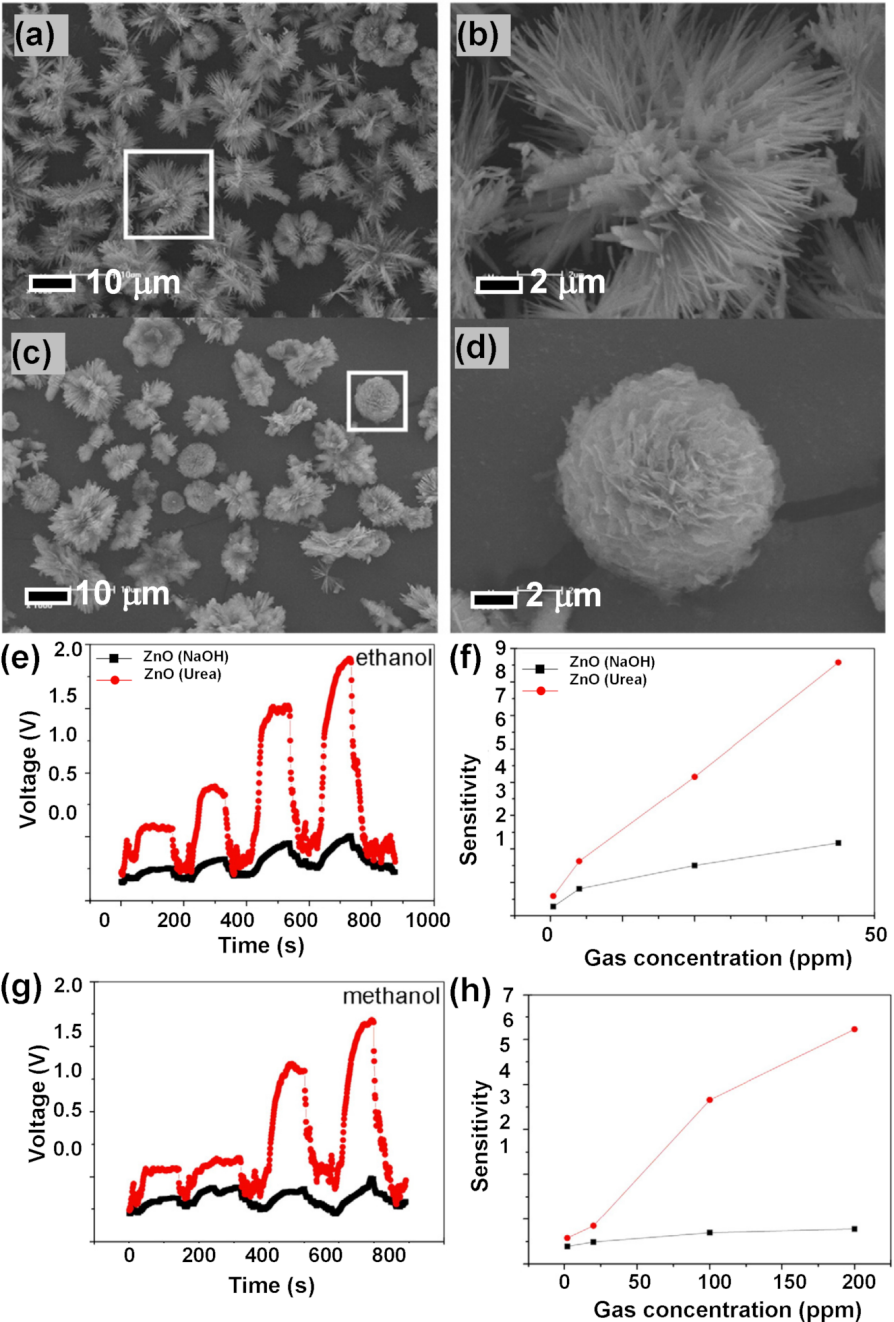
ZnO nanoscale flowers. SEM images of the samples prepared with a reaction time of (a, b) 2 h and (c, d) 6 h with a molar ratio of urea/Zn^2+^ = 2:1; (e, f) dynamic response–recovery curve and sensor sensitivity toward ethanol and (g, h) dynamic response–recovery curve and sensor sensitivity toward methanol. Figure 15a–h was reprinted from [76], Powder Technology, vol. 217, by X. Liu; J. Zhang; T. Yang; L. Wang; Y. Kang; S. Wang; S. Wu, “Self-assembled hierarchical flowerlike ZnO architectures and their gas-sensing properties”, pages no. 238–244, Copyright (2012), with permission from Elsevier. This content is not subject to CC BY 4.0.

Hierarchical dandelion-like hollow ZnO structures were reported by Fan et al. who annealed a zinc precursor [75]. Figure 14a–f shows SEM images of ZnO structures obtained at different temperatures. The fabricated structures had large surface area and affluent pores and were tested for sensing ethanol vapors. The authors reported good sensing response (34.5), rapid response (6 s), fast recovery time (7 s), and superior selectivity towards ethanol vapors at an optimum temperature of 250 °C. Figure 14g,h show the response curve, characteristic response, and recovery time for sensing 50 ppm of ethanol while operating at 250 °C. The porous dandelion-like structure enabled gas molecules to move through the abundant multiscale interconnected canals of the sensing material. Also, the large surface area of the dandelion-like structure enhanced the physical or chemical interactions due to availability of active adsorption sites at the surface of the sensing material. These structures were estimated to have *D* values in the range of 1.19–1.61. In a similar study, Liu et al. reported on flower-like ZnO hierarchical superstructures synthesized using urea through a low-temperature hydrothermal technique [76]. The obtained 3D flower-like ZnO structures had highly dendritic structures with numerous nanoscale needles. Figure 15a–d depicts SEM images of sample obtained after different reaction times and after using different concentration ratios of the precursors. Figure 15e–h illustrates sensitivity and response–recovery curves for ethanol and methanol. The 3D structures provided a large surface area while the branching of the structures helped in diffusion and transport of gas molecules within the sensing material. The samples had fractal dimensions of 1.59 (after two hours of reaction) and 1.38 (after six hours of reaction).

**Figure 16:**
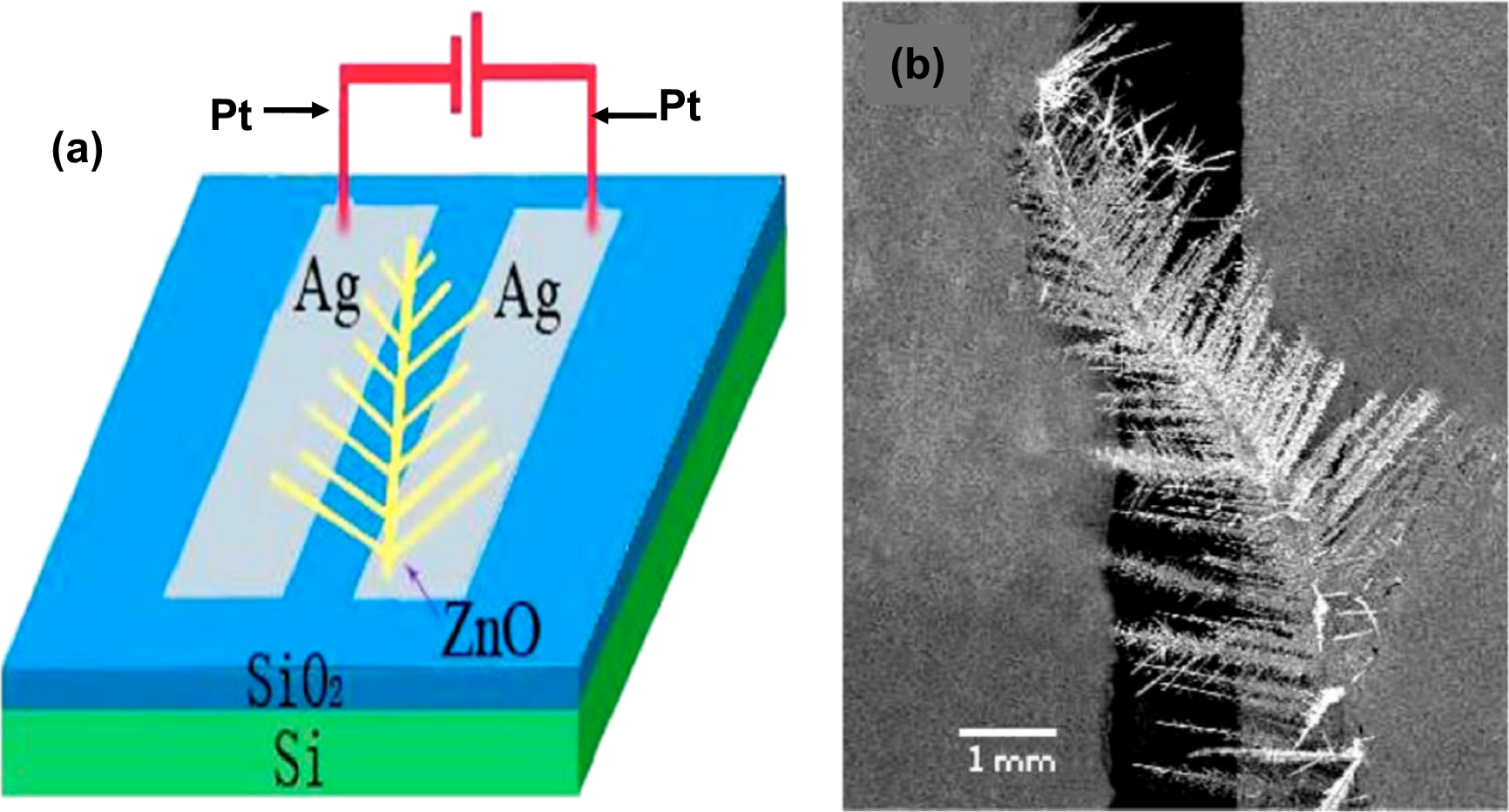
ZnO dendritic sensor. (a) Schematic illustration and (b) SEM micrograph of a ZnO dendrite gas sensor. Figure 16a,b was reprinted from [77], with the permission of AIP Publishing. This content is not subject to CC BY 4.0.

Zang et al. demonstrated the mass production of ZnO dendrites and single-crystal ZnO dendrites up to the macroscale [77]. These were synthesized via a vapor-phase transport method at 930 °C using a copper catalyst. Figure 16a and Figure 16b show, respectively, a schematic and a SEM image of the ZnO dendrite gas sensor device. The ZnO dendrites were composed of many well aligned nanorods. The variations in potential barrier height at the contacts of the nanorods gave excellent gas sensing results towards hydrogen sulfide (H_2_S). The sensitivity response of the ZnO dendrite sensor at room temperature and the variation in sensitivity at different H_2_S concentrations (10–500 ppm) was studied. For 10 ppm the sensitivity of the sensor was observed to be 3.3 while that for 500 ppm was 26.4. The response time for the dendritic sensors was observed to be in the range of 15–20 s, and the sensors recovered in 30–50 s. These structures show a fractal dimension of 1.79.

#### Tungsten oxide-based fractals

**Figure 17:**
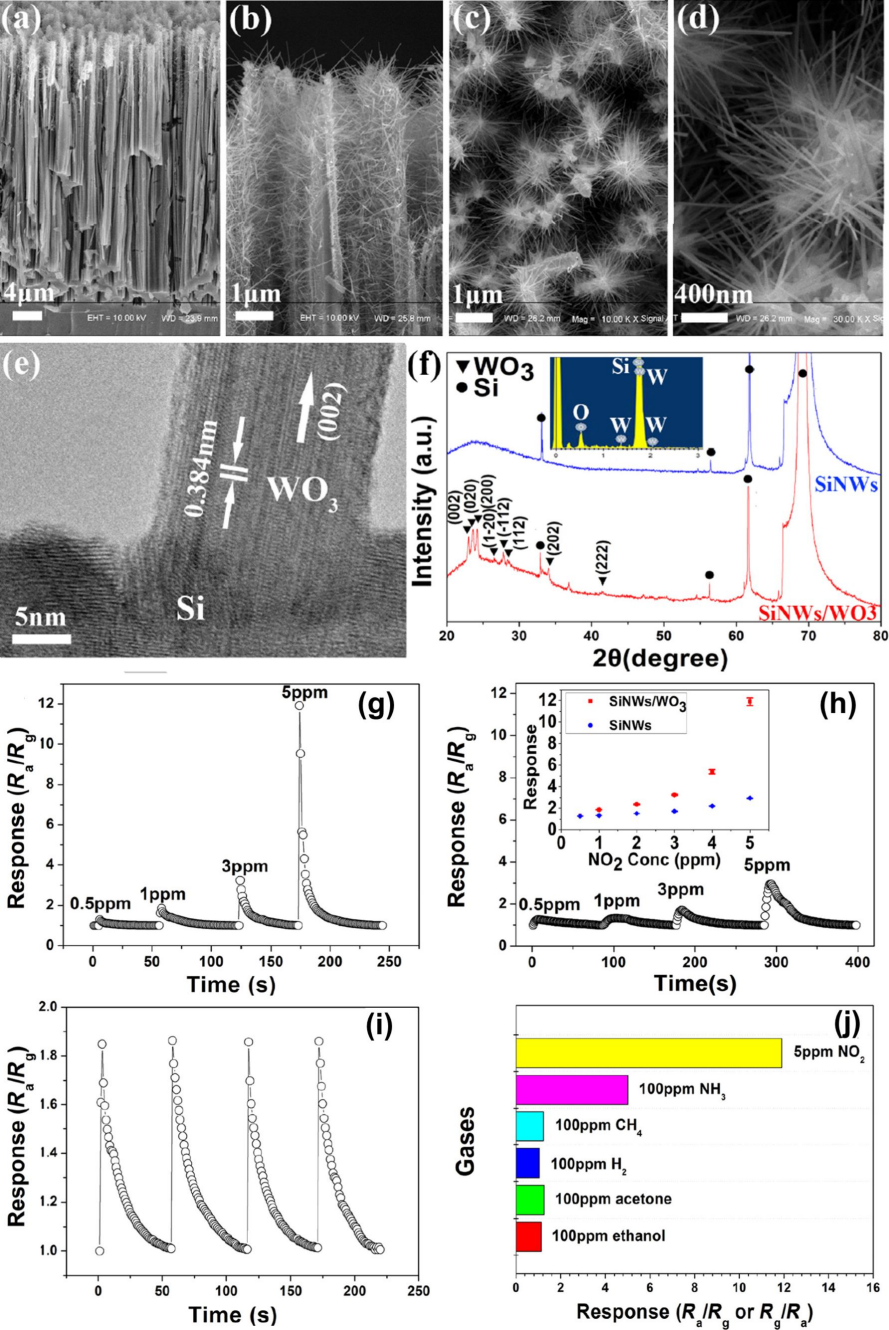
Si/WO_3_ nanowires. (a–d) SEM images of Si/WO_3_ NWs, (e) HRTEM image of a WO_3_/SiNW interface, (f) XRD pattern of SiNWs and SiNWs/WO_3_. Dynamic responses of (g) the composite and (h) pure SiNWs to 0.5–5 ppm NO_2_ at room temperature; (i) four cycles of dynamic response of the composite sensor to 1 ppm NO_2_; (j) response of the composite sensor to different gases. The inset in (h) shows the response values at different NO_2_ concentrations. Figure 17a–j was reprinted from [79], Materials Letters, vol. 207, by Y. Qin; Z. Wang; D. Liu; K. Wang, “Dendritic composite array of silicon nanowires/WO_3_ nanowires for sensitive detection of NO_2_ at room temperature”, pages no. 29–32, Copyright (2017) with permission from Elsevier. This content is not subject to CC BY 4.0.

**Figure 18:**
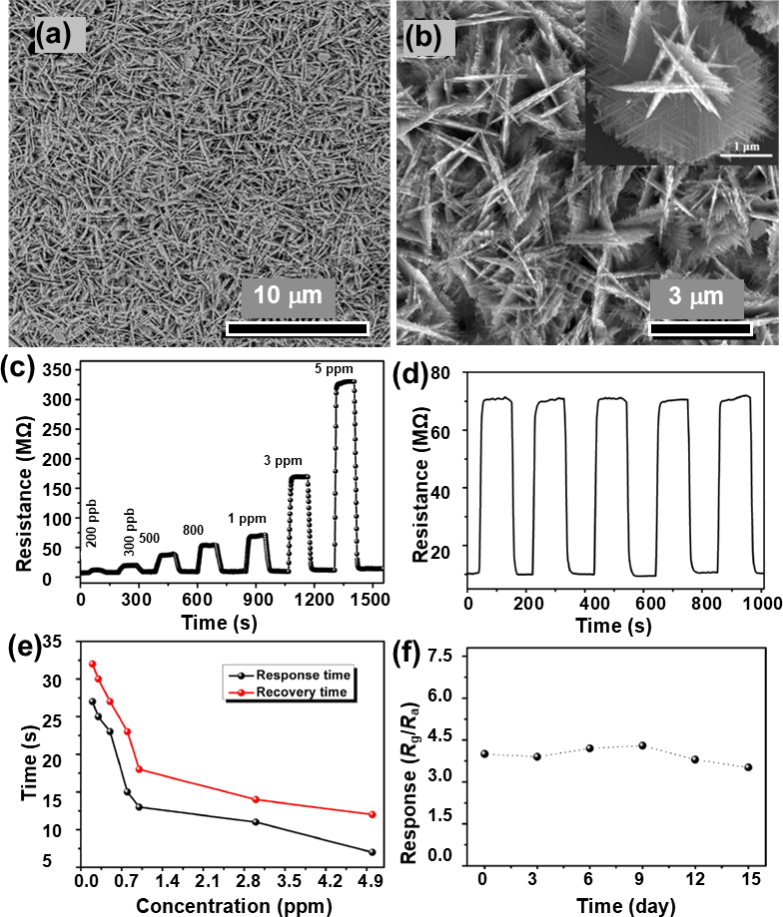
WO_3_ dendrites. (a, b) SEM images of the grown hierarchical WO_3_ dendrites at low and high magnification; (c) dynamic response curves of the WO_3_ sensor as function of the NO_2_ concentration; (d) typical response curve cycling to 500 ppb of NO_2_ at 140 °C; (e) variations in response and recovery times at 140 °C as function of the NO_2_ concentration; (f) stability study of the sensor exposed to 500 ppb NO_2_ at 140 °C. Figure 18a–f were reprinted from [80], Ceramics International, vol. 43, by B. Xiao; D. Wang; F. Wang; Q. Zhao; C. Zhai; M. Zhang, “Preparation of hierarchical WO_3_ dendrites and their applications in NO_2_ sensing”, pages no. 8183–8189, Copyright (2017), with permission from Elsevier. This content is not subject to CC BY 4.0.

A very recent study on the sensing of NO_2_, acetone, and carbon monoxide was reported by Simon and co-workers. They used Ni nanoparticles to decorate a reduced graphene oxide/WO_3_ nanocomposite [78]. The WO_3_ sample annealed at 600 °C shows the presence of fractal structures. Though the authors of the work did not consider the formation of fractals in their work, the SEM image of the sample annealed at 600 °C was used to estimate the fractal dimension. The analysis shows that this particular sample had a fractal dimension of *D* = 1.86. Metal-assisted chemical etching was used by Qin et al. [79] to prepare a dendritic array of Si/WO_3_ NW composites, which was tested for the detection of NO_2_ gas at room temperature. Figure 17a–e SEM and high-resolution transmission electron microscopy (HR-TEM) images of Si/WO_3_ NWs. Figure 17f shows the XRD diffractograms of Si NWs and Si/WO_3_ NWs. Figure 17g–j demonstrates the dynamic response of composite and pure Si NWs to NO_2_ at different concentrations at room temperature, and the response of the composite to different gases. The composite sensor with p–n heterojunctions successfully transferred charge carriers and additionally served as the conduction path for electron transportation, which led to an improvement in gas sensing behavior of the composite sensor. The technique yielded structures with a fractal dimension of 1.73. In another study, NO_2_ sensing by WO_3_ dendritic nanosheets, prepared by Xiao et al. using a solvothermal method, was reported [80]. Here, the authors discussed a five-stage growth process comprising polymerization, nucleation, primary growth, secondary growth, and final growth from single nanosheets to final dendritic structures. Figure 18a and Figure 18b show SEM images of hierarchical WO_3_ dendrites at different magnifications. The nanostructured dendrites exhibited a higher sensitivity with a detection limit of 200 ppb towards NO_2_, with rapid response (7 s) and recovery time (12 s) at 5 ppm NO_2_ at an operating temperature of 140 °C. Figure 18c shows the response curves (at 140 °C) of the WO_3_ sensor to NO_2_. Figure 18d shows the resistance as function of the time, Figure 18e shows the response and recovery times as functions of the NO_2_ concentration, and Figure 18f demonstrates the stability of the response towards 500 ppb of NO_2_ for up to 15 days. The authors observed that ethanol and citric acid played a vital role in the growth of the dendrite nanostructure, which exhibited a fractal dimension of 1.94.

#### Bismuth vanadate -based fractals

**Figure 19:**
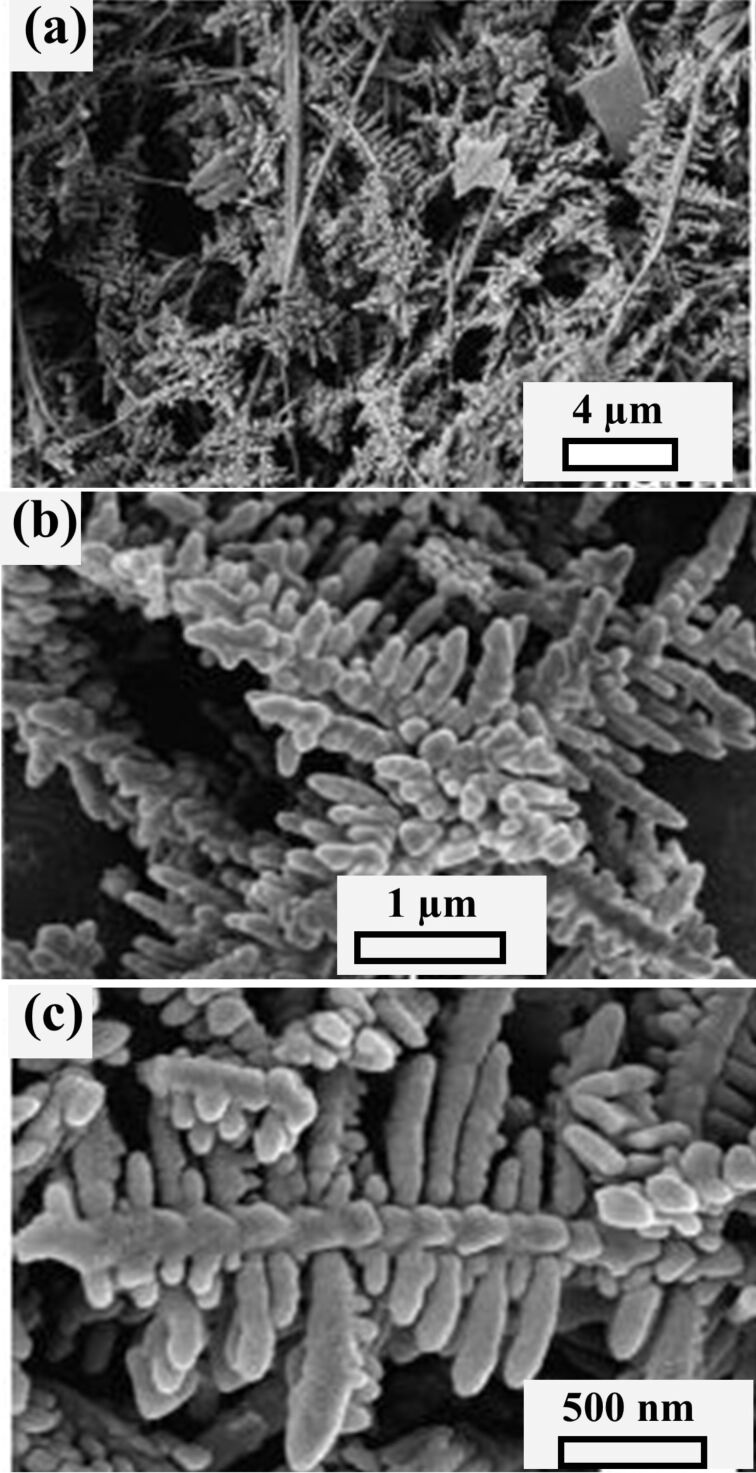
Bismuth vanadate hyperbranched structures. (a) FESEM image of hyperbranched m-BiVO_4_; (b) magnified FESEM image that shows a single hyperbranch of h-BiVO_4_; (c) magnified FESEM image that shows the trunk of h-BiVO_4_. Figure 19a–c are reproduced from [81], Y. Zhao, Y. Xie, X. Zhu, S. Yan, S. Wang, “Surfactant-Free Synthesis of Hyperbranched Monoclinic Bismuth Vanadate and its Applications in Photocatalysis, Gas Sensing, and Lithium-Ion Batteries”, Chemistry – A European Journal, with permission from John Wiley & Sons. Copyright © 2008 WILEY-VCH Verlag GmbH & Co. KGaA, Weinheim. This content is not subject to CC BY 4.0.

**Figure 20:**
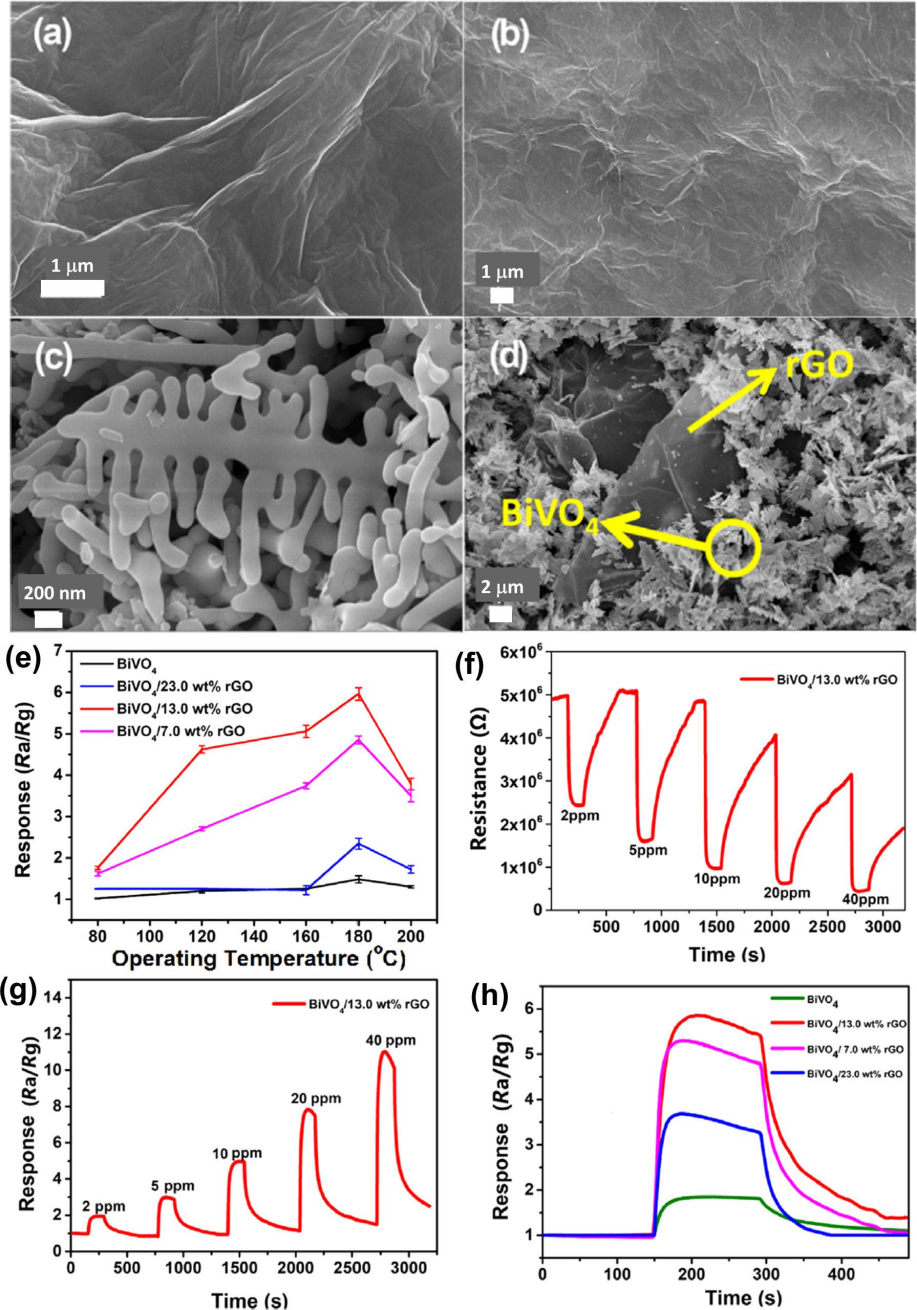
Bismuth vanadate dendrites. SEM images of (a) GO, (b) rGO, (c) pure pine dendritic BiVO_4_, and (d) BiVO_4_/rGO hybrid; (e) response of pure BiVO_4_ and BiVO_4_/rGO hybrids towards 10 ppm TEA at different operating temperatures and 35% relative humidity; (f, g) resistance and response curves for a sensor based on BiVO_4_/13.0 wt% rGO to different concentrations of TEA at 180 °C; (h) response of pure BiVO_4_ and BiVO_4_/rGO hybrids towards 10 ppm TEA and 35% relative humidity. Figure 20a–h was reprinted from [82], Journal of Colloid and Interface Science, vol. 587, by S. Bai; L. Sun; J. Sun; J. Han; K. Zhang; Q. Li; R. Luo; D. Li; A. Chen, “Pine dendritic bismuth vanadate loaded on reduced graphene oxide for detection of low concentration trimethylamine”, pages no. 183–191, Copyright (2021), with permission from Elsevier. This content is not subject to CC BY 4.0.

Zhao et al. synthesized large-scale highly uniform hyperbranched monoclinic BiVO_4_ (h-BiVO_4_) structures by a surfactant-free hydrothermal method [81]. The as-prepared h-BiVO_4_ structure exhibited high sensitivity towards formaldehyde and ethanol. The formation of hyperbranched structures was found to be a function of different pH values, proton intercalation, and dissolution processes. The sensitivity in case of h-BiVO_4_ was found to be excellent as compared to monoclinic bismuth vanadate (m-BiVO_4_) at room temperature owing to the hyperbranched structure and high surface area. Figure 19a–c shows field-emission SEM (FESEM) images of hyperbranched m-BiVO_4_, a single hyperbranch of h-BiVO_4_, and a trunk of h-BiVO_4_. Recently, Bai et al. used hydrothermal method to synthesize reduced graphene oxide and pine dendritic BiVO_4_ composite with an average length of 1–1.5 μm and about 0.6 μm width [82]. In the hybrid composite rGO nanosheets were draped with a pine dendritic morphology. Figure 20 shows the SEM images of GO (Figure 20a), rGO (Figure 20b), pure pine dendritic BiVO_4_ (Figure 20c), and the BiVO_4_/rGO hybrid structure (Figure 20d). The hybrid material was used for the detection of triethylamine (TEA) gas. A detection of 10 ppm TEA with the highest response (5.91) was achieved with the hybrid composition of BiVO_4_ and rGO at 180 °C working temperature, in comparison to pure BiVO_4_ (1.2) and other compositions of BiVO_4_ and rGO at different temperatures (80–200 °C). The outstanding enhancement in the response of the hybrid material with quick response and recovery times was attributed to the formation of p–n heterojunctions between rGO nanosheets and dendritic BiVO_4_, the increased surface area of dendritic structures, as well as conductivity and acceleration of electrons between gas molecules and hybrid material. Figure 20e shows responses of pure BiVO_4_ and BiVO_4_/rGO hybrids towards 10 ppm TEA with different rGO mass ratios at different temperatures. Figure 20f and Figure 20g show, respectively, resistance and response curves for the sensor based on the BiVO_4_/13.0 wt % rGO hybrid material to different concentrations of TEA at 180 °C. Figure 20h illustrates responses of sensors based on pure BiVO_4_ and the BiVO_4_/rGO hybrid material towards 10 ppm TEA at 35% RH.

#### Cadmium sulfide-based fractals

**Figure 21:**
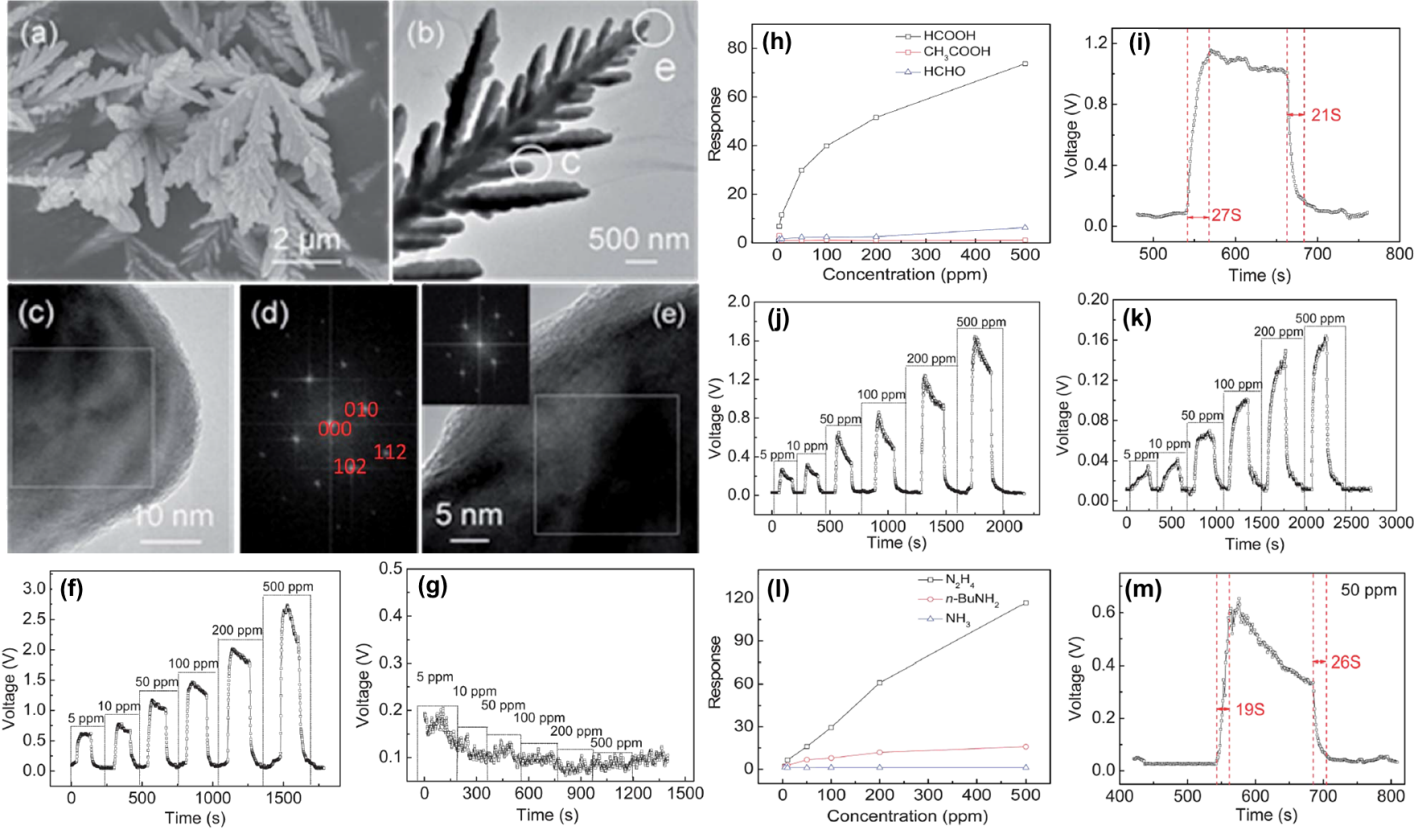
CdS dendrites. (a, b) CdS dendrites observed under SEM and TEM. The marked regions “c” and “e” were explored further; (c) HR-TEM and (d) FFT images of the region marked as “c”; (e) HR-TEM and (f) and FFT images of the region marked as “e”. (f, g) Dynamic response–recovery curves of the sensor to formic and acetic acid vapors at different concentrations at 260 °C, respectively; (h) response as function of the gas concentration of the HOCSD sensor; (i) response transients of the sensor to 50 ppm HCOOH at 260°C; (j, k) dynamic response–recovery curves of the sensor to N_2_H_4_ and *n*-BuNH_2_ at different concentrations at 260°C, respectively; (l) response as function of the gas concentration of the HOCSD sensor; (m) response transients of the sensor to 50 ppm N_2_H_4_ at 260 °C. Figure 21a–m was republished with permission of The Royal Society of Chemistry from [83] (“Synergistic effect of the reducing ability and hydrogen bonds of tested gases: highly orientational CdS dendrite sensors” by W. Guo et al., J. Mater. Chem. A, vol. 2, issue 4, © 2014); permission conveyed through Copyright Clearance Center, Inc. This content is not subject to CC BY 4.0.

Highly oriented CdS dendrite (HOCSD) sensors synthesized by a hydrothermal method to detect formic acid (HCOOH) and hydrazine (N_2_H_4_) were reported by Guo and co-workers [83]. The multichannel branches of dendritic structure of the CdS sensor allowed gas molecules to penetrate the sensor more easily. The response and recovery times for a small concentration of HCOOH (ca. 50 ppm) was reported to be ca. 27 s and 21 s at 260 °C, respectively. Figure 21a–e shows SEM, TEM and HR-TEM images of the CdS dendrites. The CdS dendrites were shown to have superior diffusion and adsorption/desorption properties. Also, there was a synergistic effect of hydrogen bond formation and reducing abilities of the tested gas. Figure 21f and Figure 21g illustrate response and recovery time curves of the sensor when exposed to vapors of formic and acetic acid at 260 °C, respectively. Figure 21h and Figure 21i show the response as function of the concentration of the HOCSD sensor and response transients towards 50 ppm of HCOOH at 260 °C, respectively. Figure 21j and Figure 21k show the dynamic response curve for N_2_H_4_ and *n*-BuNH_2_ with varying concentrations at an operating temperature of 260 °C, while Figure 21l and Figure 21m show the response towards 50 ppm of N_2_H_4_ at 260 °C.

#### Other oxide-based fractals

**Figure 22:**
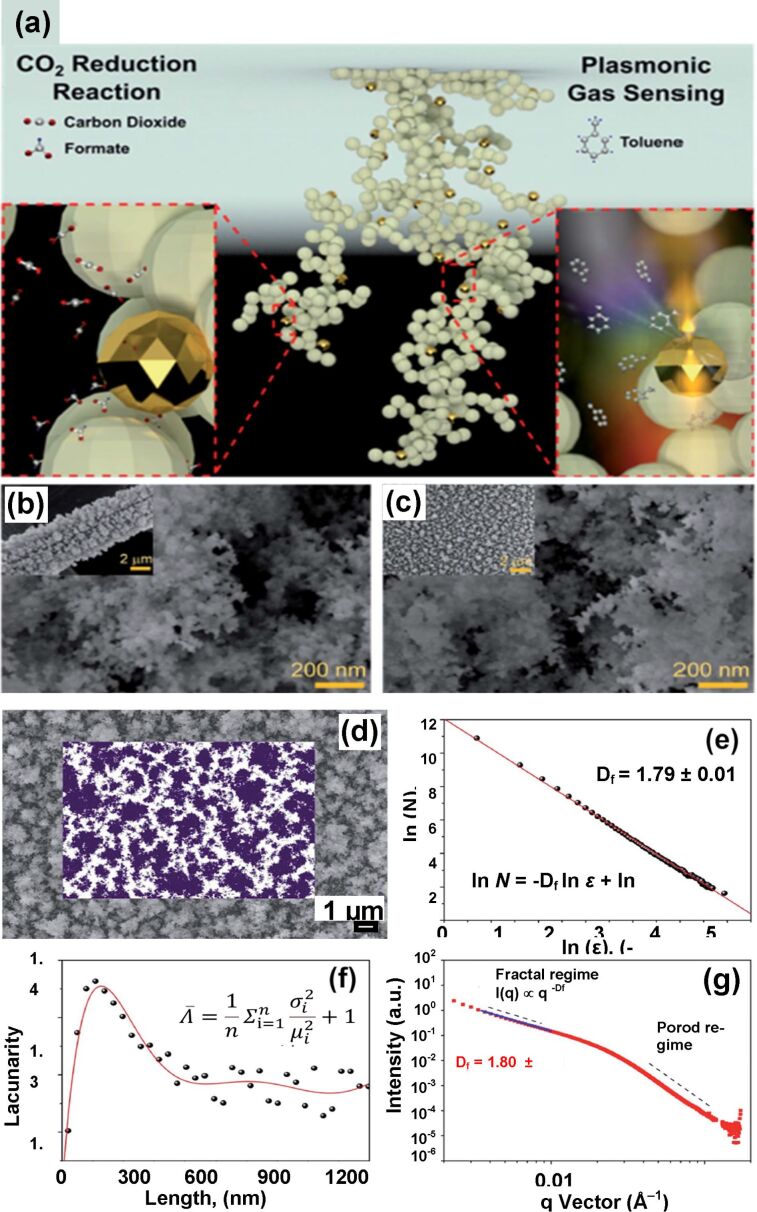
Au–Bi_2_O_3_ fractals. (a) Schematic of a Au–Bi_2_O_3_ fractal shown with magnified regions showing reduction of CO_2_ to formate (left) and optical gas sensing of toluene (right); (b, c) SEM images of the sample on carbon fiber paper and glass, respectively; (d) SEM image of Au–Bi_2_O_3_ (inset: fractal analysis using the box-counting method); (e) fractal dimension of 1.79; (f) lacunarity of the Au–Bi_2_O_3_ film. (g) Double-log plot of scattering intensity and vector *q* measured by small angle X-ray scattering of the Au–Bi_2_O_3_ film on a glass substrate. Figure 22a–g was republished with permission of The Royal Society of Chemistry from [45] (“Multifunctional nanostructures of Au–Bi_2_O_3_ fractals for CO_2_ reduction and optical sensing” by T. Tran-Phu et al., J. Mater. Chem. A, vol.8, issue 22, © 2020); permission conveyed through Copyright Clearance Center, Inc. This content is not subject to CC BY 4.0.

In 2020, Tran-Phu et al. demonstrated the formation of three-dimensional fractals of Au–Bi_2_O_3_, having *D* ≈ 1.80, on a substrate by hot-aerosol synthesis [45]. The fabricated Au–Bi_2_O_3_ porous fractal structures contained abundant active sites for the adsorption of carbon dioxide and other VOCs. An improvement of the electron density was attributed to gold nanoparticles. The resulting fractal structures showed excellent sensing properties towards VOCs (100 ppm at room temperature). The samples were used in the electrochemical reduction of carbon dioxide and for an optical sensor based on LSPR. Figure 22a shows the Au–Bi_2_O_3_ fractal structures and the optical sensing of formate and toluene. Figure 22b and Figure 22c show SEM images of the sample on carbon fiber paper and a glass substrate, respectively. Figure 22d illustrates the SEM image of Au–Bi_2_O_3_ fractal employed for box-counting. Figure 22e–g shows the estimation of the fractal dimensions of the fabricated cluster.

**Figure 23:**
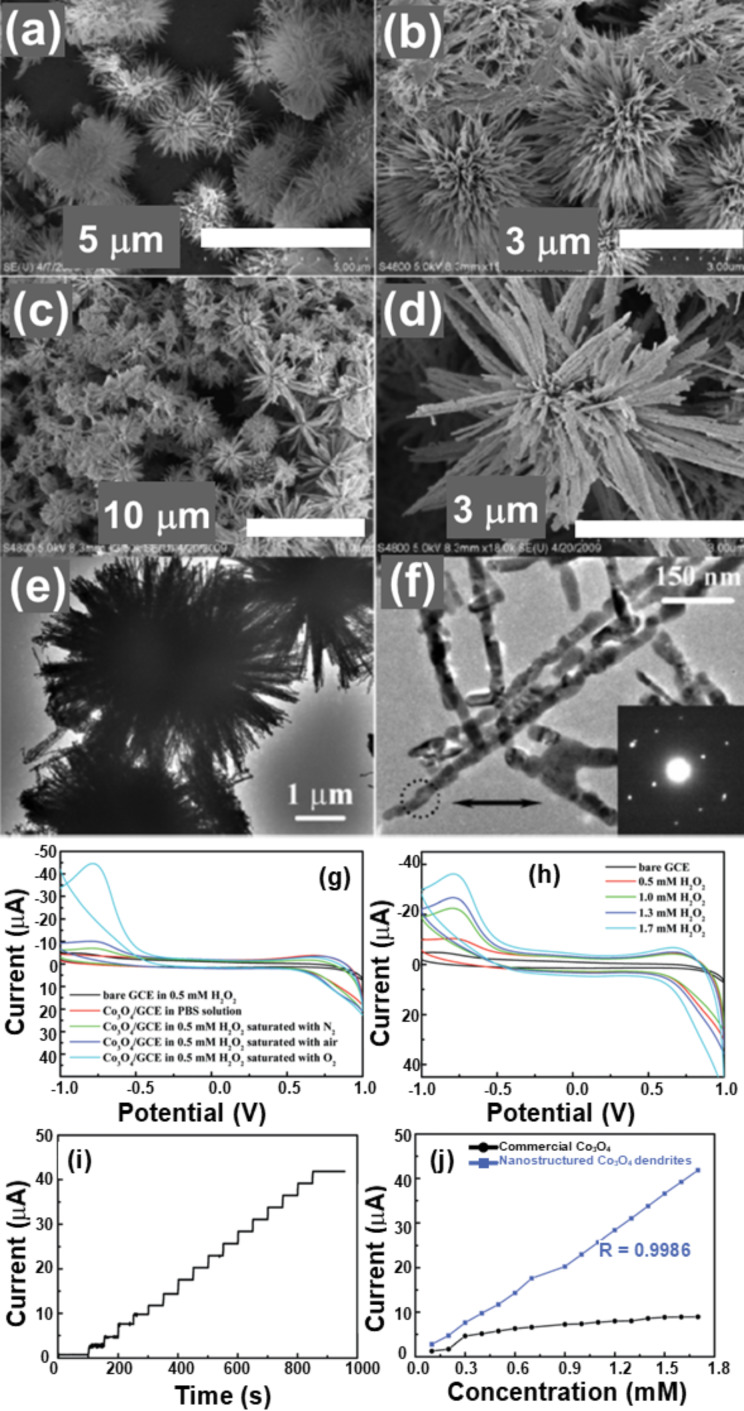
Co_3_O_4_ dendrites. SEM images of (a, b) precursor and (c, d) Co_3_O_4_ nanostructure; (e, f) TEM images of the Co_3_O_4_ nanostructure (inset in (f): SAED pattern of the encircled area); (g) CV curves of a bare glassy carbon electrode (GCE) and a GCE modified with the as-prepared Co_3_O_4_ nanostructure recorded in 0.05 M PBS (pH 7.40) solution saturated with different gases; (h) CV curves of a GCE modified with Co_3_O_4_ nanostructures at different concentrations of H_2_O_2_ saturated with N_2_ recorded in 0.05 M PBS (pH 7.40); (i) amperometry response at −0.77 V to successive increments of H_2_O_2_ concentration; (j) current as function of the H_2_O_2_ concentration measured with different Co_3_O_4_-modified GCEs. Figure 23a–j was republished with permission of The Royal Society of Chemistry from [84] (“Dendrite-like Co_3_O_4_ nanostructure and its applications in sensors, supercapacitors and catalysis”, H. Pang et al., Dalton Trans., vol. 41, issue19, © 2012); permission conveyed through Copyright Clearance Center, Inc. This content is not subject to CC BY 4.0.

Pang et al. synthesized a dendrite-like Co_3_O_4_ nanostructure composed of numerous nanorods (15–20 nm diameter and 2–3 µm length) by a hydrothermal method and calcined the fabricated nanostructure precursors in air [84]. Figure 23a–f shows SEM and TEM micrographs with selected-area electron diffraction (SAED) patterns of the Co_3_O_4_ nanostructures. Hydrogen peroxide (H_2_O_2_) was detected by an electrochemical sensor based on the Co_3_O_4_ fractals. The results confirmed that the Co_3_O_4_ dendritic sensor exhibited a higher sensitivity than a commercial Co_3_O_4_ sensor. Figure 23g–j show the H_2_O_2_ detection results. The fractals were estimated to have a fractal dimension of 1.74. Wang et al. [85] synthesized a Christmas tree-like structure of nanoscale Zn-doped nickel oxide dendritic crystals by an electrolytic method with high-temperature oxidation for the detection of NH_3_ at room temperature. Zn-doped NiO dendritic crystals at the nanoscale consisted of a major elongated stem having numerous secondary and tertiary branches. The dendritic nanostructure allowed the network passage for electron transfer after ammonia molecules interact with the sensing surface. It showed an about 5–8 times enhanced response and an improvement in recovery time by about 30–50 times compared to a pristine NiO sensor. The sensor also showed good reproducibility, high stability, and selectivity towards ammonia over other gases.

In 2015, Zhao et al. reported dandelion-like NiO hierarchical structures assembled with dendritic elements (ca. 1.8 µm) synthesized via a surfactant-free one-step hydrothermal route [86]. The dandelion-like NiO hierarchical structures had an incredibly rough surface and many gaps among them. These structures demonstrated a good response to 100 ppm ethanol with very quick response (2 s) and recovery time (12 s) at 240 °C. The better sensitivity and faster response and recovery times were due to the hierarchical structure, inherent rough surfaces, and gaps acting as diffusion channels.

### Gas-sensing mechanism of fractal structures

There are a number of models to explain the function of conductometric SMO gas sensors. For instance, electron depletion layer (for n-type materials) or hole accumulation layer theory (for p-type), fermi level control theory, and grain boundary barrier control theory models have been proposed to understand the fundamentals of sensing mechanism [31,36,87–88]. The changes in electrical resistance of materials from a microscopic viewpoint are addressed by electronic and chemical sensitization mechanisms [26]. The mechanisms focusing on a macroscopic perspective deal with adsorption/desorption of surface atomic/molecular species or discuss how the bulk resistance and gas diffusion control mechanisms affect the charge transport [26,87,89]. The changes in electrical resistance of the material upon interaction with target gas analytes arise due to changes in, for instance, energy bands, surface charge, and work function caused by temperature, grain size, crystal plane energies, and doping [87,90–91]. Thus, there is no single sensing mechanism that explains all SMO gas sensors. The section “Fractal length scales and growth models” described various fab-fracs tested as gas sensors for different analytes. The role of the morphology of fab-fracs in the gas sensing response will now be discussed.

**Figure 24:**
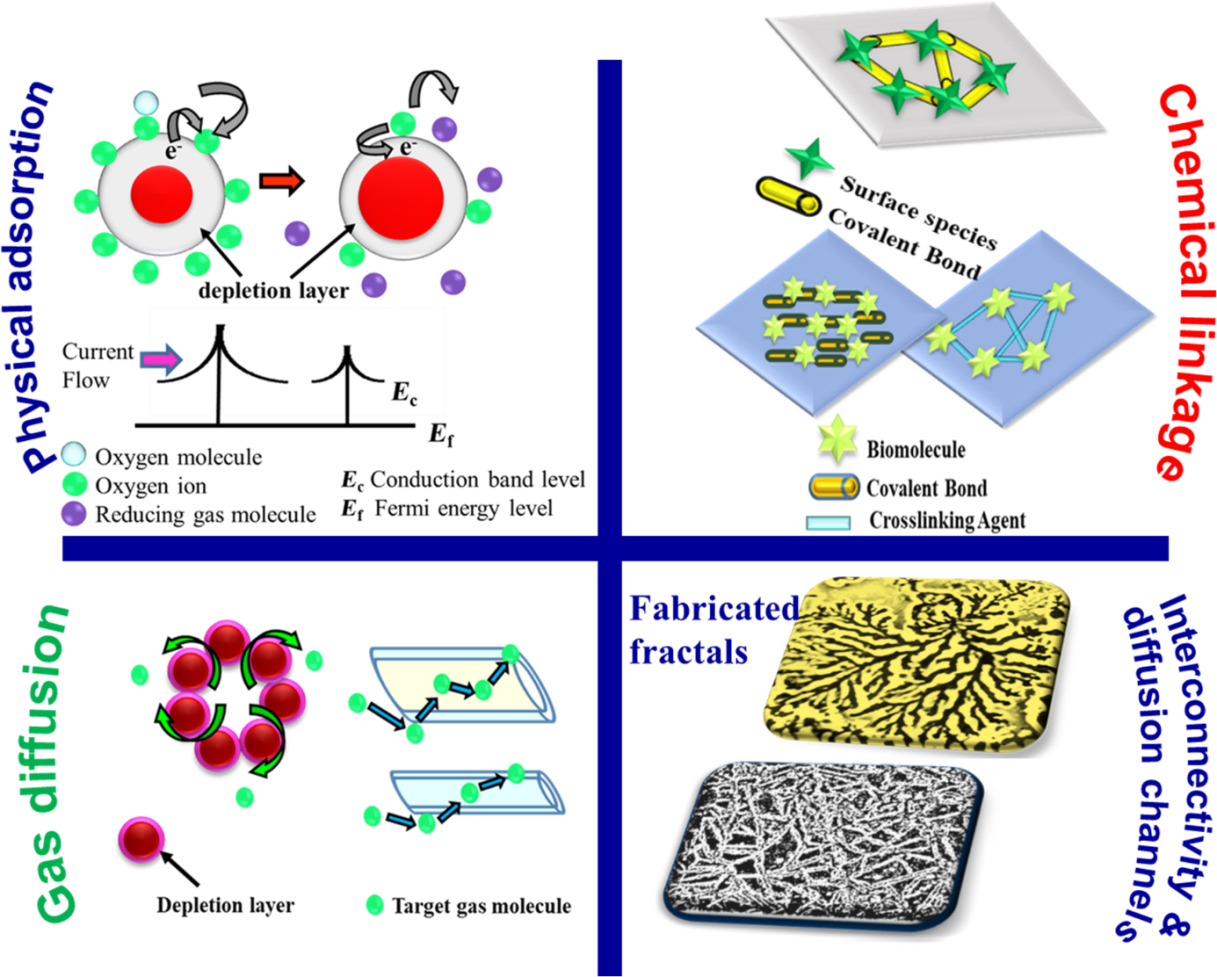
Gas sensing mechanisms. The schematic presents the different mechanisms through which SMO sensors can interact with analyte gases. The pore network, channel interconnectivity, and crossover points in case of fab-fracs are also shown.

Figure 24 explains the common mechanisms of physical adsorption, chemical linkages, gas diffusion, and fractal interconnectivity and channels. It is known that nanocrystalline gas sensor materials have better sensitivity because of their large surface area [92]. Fractal structures have dimensions that can range from micrometers to millimeters, but they primarily are composed of nanostructures that have aligned themselves in some specific way guided by surface diffusion during their growth. The transition of a fractal structure from one length-scale to another is the result of self-organization and/or re-organization process. Since fractals grow primarily via diffusion, the network is usually always continuous thereby serving as an underlying porous network decorated with three-dimensional unique geometric structures. The fab-frac structures comprise building blocks at different length scales of varying sizes and orientations. The abundant accessibility of intercrossing and interconnections of these building blocks with each other result in the formation of 3D porous network structures. This increases coarsening, roughness, and adsorption sites and overall offers a high surface-to-volume ratio [72,82,91]. The crossovers offer additional secondary and tertiary adsorption sites. Thus, the extension of fractals in all three dimensions not only adds to the total number of active adsorption sites but also enhances their density. It is well known that surface gas adsorption and desorption are the rate-determining steps and mostly depend on surface characteristics such as surface area, roughness, porosity, branching, network structure, and fractal dimensions. The fractal dimensions estimated in the present article show that structures with *D* in the range of 1.3–1.8 exhibit better gas sensing responses. For fractal dimensions greater than 2, gas sensing behavior is not significant, and this could be due to grain boundary resistance, which controls the charge transport and thereby nullifies the effect of fractal morphology. Further, the possibility of gas molecules to diffuse in the material via surface diffusion, Knudsen diffusion (radius of pores in the range of 1–100 nm) and molecular diffusion (radius of pores greater than 100 nm) is higher. Fractals offer all three possibilities. The different size of pores (macropores with pore sizes above 50 nm and mesopores with pore sizes in the range of 2–50 nm) are associated with different functions such as delivery, withdrawal reaction canals, and centers for adsorptions [57,69]. For larger pore sizes, rapid gas diffusion rates are observed leading to higher values of sensitivity [26,89]. Thus, while the porous continuous network provides a backbone for better and faster charge transport, the unique morphology of fab-fracs offers a better gas–sensor interaction indicated by the fractal dimension.

## Conclusion

Fractals are intriguing structures that are crafted by natural processes and can be fabricated in labs in a controlled environment. Their unique morphologies comprise structures ranging from the nanoscale to the macroscale, where the properties change with the length scales involved. This aspect has been addressed in the present review with focus on gas-sensing properties. While chemical or physical properties of the material may or may not remain the same, a change in geometry and architecture, especially as fractals, can aid in better sensing. Connectivity, exposure to gaseous environment, nanoscale 3D coarsening, and roughness generate many secondary and tertiary adsorption sites that result in better sensitivity. If somehow the interconnectivity can be improved with the help of an artificial electrode, better sensing characteristics can be expected. Thus, fractal geometries show great prospect to be considered as sensor templates, regardless of the used material. The nucleation, growth, and diffusion-limited aggregation of nanoparticles results in the formation of interesting fractal morphologies with high surface-to-volume ratio, high porosity, and interconnectivity within branched structures. Different fractal morphologies with empirically found fractal dimensions of 1.5–1.86 have shown better sensing results. Therefore, studies can be planned to focus on the fractal dimension by theoretical and experimental approaches. Here, lithography techniques can be implemented to write fractals of different fractal dimensions and their response under identical test conditions can be studied. Such structures can also be explored as substrates for surface-enhanced Raman spectroscopy, which finds applications in areas ranging from food packaging to medical diagnosis. Fractals can also help in mimicking the wound healing process as tissue grows to connect the torn skin across the wound and offer an insight into microfluids as part of wound healing management. Besides application-oriented research the hyperbranched morphologies of fractals, offering high surface area and numerous transport channels for gas analytes to reach the electrode of a sensor more quickly, also form a good object of basic science. This review article gives an overview of fractal geometries that have been successfully applied as gas-sensing elements, shows the possibility of growing fab-fracs under controlled lab conditions, and opens a number of ideas that can be taken up by researchers. Indeed, there is little known in the world of fractals and a lot to explore and learn.

## Supporting Information

File 1 Summary of the fractal structures and their applications.

## References

[1] Smith H O (1937). The London, Texas, School Disaster. The Quarterly of the National Fire Protection Association.

[2] Broughton E (2005). Environ Health (London, U K).

[3] (2021). 500 on 2 Trains Reported Killed By Soviet Gas Pipeline Explosion.

[4] (2020). India gas leak: At least 11 dead after Visakhapatnam incident.

[5] Chen Y, Zhang W, Wu Q (2017). Sens Actuators, B.

[6] Kolmakov A, Moskovits M (2004). Annu Rev Mater Res.

[7] Korotcenkov G (2007). Mater Sci Eng, B.

[8] Yunusa Z, Hamidon M N, Kaiser A, Awang Z (2014). Sens Transducers J.

[9] Wang C, Yin L, Zhang L, Xiang D, Gao R (2010). Sensors.

[10] Thong L V, Loan L T N, Van Hieu N (2010). Sens Actuators, B.

[11] Tonezzer M (2019). Sens Actuators, B.

[12] Brunet E, Maier T, Mutinati G C, Steinhauer S, Köck A, Gspan C, Grogger W (2012). Sens Actuators, B.

[13] Chen X, Shen Y, Zhang W, Zhang J, Wei D, Lu R, Zhu L, Li H, Shen Y (2018). Appl Surf Sci.

[14] Liang J, Zhao Y, Zhu K, Guo J, Zhou L (2019). Thin Solid Films.

[15] Yu H Y, Kang B H, Pi U H, Park C W, Choi S-Y, Kim G T (2005). Appl Phys Lett.

[16] Thai N X, Van Duy N, Hung C M, Nguyen H, Tonezzer M, Van Hieu N, Hoa N D (2020). J Sci: Adv Mater Devices.

[17] Xu J, Wang D, Qin L, Yu W, Pan Q (2009). Sens Actuators, B.

[18] Jagadale S B, Patil V L, Vanalakar S A, Patil P S, Deshmukh H P (2018). Ceram Int.

[19] Kwon D-K, Porte Y, Ko K Y, Kim H, Myoung J-M (2018). ACS Appl Mater Interfaces.

[20] Dhayal Raj A, Pazhanivel T, Suresh Kumar P, Mangalaraj D, Nataraj D, Ponpandian N (2010). Curr Appl Phys.

[21] Lou Z, Wang L, Wang R, Fei T, Zhang T (2012). Solid-State Electron.

[22] Li Z, Wang N, Lin Z, Wang J, Liu W, Sun K, Fu Y Q, Wang Z (2016). ACS Appl Mater Interfaces.

[23] Liu Y, Jiao Y, Zhang Z, Qu F, Umar A, Wu X (2014). ACS Appl Mater Interfaces.

[24] Liu J, Wang X, Peng Q, Li Y (2005). Adv Mater (Weinheim, Ger).

[25] Kolmakov A, Klenov D O, Lilach Y, Stemmer S, Moskovits M (2005). Nano Lett.

[26] Wang X, Wang Y, Tian F, Liang H, Wang K, Zhao X, Lu Z, Jiang K, Yang L, Lou X (2015). J Phys Chem C.

[27] Zhou X, Liu J, Wang C, Sun P, Hu X, Li X, Shimanoe K, Yamazoe N, Lu G (2015). Sens Actuators, B.

[28] Park H J, Choi N-J, Kang H, Jung M Y, Park J W, Park K H, Lee D-S (2014). Sens Actuators, B.

[29] Nazemi H, Joseph A, Park J, Emadi A (2019). Sensors.

[30] Lee J-H (2009). Sens Actuators, B.

[31] Kim H-J, Lee J-H (2014). Sens Actuators, B.

[32] Goldoni A, Alijani V, Sangaletti L, D'Arsiè L (2018). Electrochim Acta.

[33] Walker J M, Akbar S A, Morris P A (2019). Sens Actuators, B.

[34] Miller D R, Akbar S A, Morris P A (2014). Sens Actuators, B.

[35] Franke M E, Koplin T J, Simon U (2006). Small.

[36] Hung C M, Le D T T, Van Hieu N (2017). J Sci: Adv Mater Devices.

[37] Dey A (2018). Mater Sci Eng, B.

[38] Sun D, Luo Y, Debliquy M, Zhang C (2018). Beilstein J Nanotechnol.

[39] Mandelbrot B B, Freeman W H (1983). The Fractal Geometry of Nature.

[40] Yang T, Tian F, Covington J A, Xu F, Xu Y, Jiang A, Qian J, Liu R, Wang Z, Huang Y (2019). Chemosensors.

[41] Wang Z, Liu T, Jiang L, Asif M, Qiu X, Yu Y, Xiao F, Liu H (2019). ACS Appl Mater Interfaces.

[42] Cao M, Liu T, Gao S, Sun G, Wu X, Hu C, Wang Z L (2005). Angew Chem, Int Ed.

[43] Chen Z, Pan D, Zhao B, Ding G, Jiao Z, Wu M, Shek C-H, Wu L C M, Lai J K L (2010). ACS Nano.

[44] Kandjani A E, Sabri Y M, Field M R, Coyle V E, Smith R, Bhargava S K (2016). Chem Mater.

[45] Tran-Phu T, Daiyan R, Fusco Z, Ma Z, Abd Rahim L R, Kiy A, Kluth P, Guo X, Zhu Y, Chen H (2020). J Mater Chem A.

[46] Townsley M I (2012). Compr Physiol.

[47] (2020). SnowCrystals.com.

[48] Ng T F, Teh G H (2009). Bull Geol Soc Malays.

[49] Fusco Z, Rahmani M, Bo R, Verre R, Motta N, Käll M, Neshev D, Tricoli A (2018). Adv Mater (Weinheim, Ger).

[50] Plugotarenko N K, Petrov V V, Ivanetz V A, Smirnov V A (2011). Glass Phys Chem.

[51] Sahoo P, Sairam G M, Kamruddin M, Dash S, Tyagi A (2017). Adv Mater (Weinheim, Ger).

[52] Witten T A, Sander L M (1981). Phys Rev Lett.

[53] Vicsek T (1984). Phys Rev Lett.

[54] Nagar R, Teki R, Koratkar N, Sathe V G, Kanjilal D, Mehta B R, Singh J P (2010). J Appl Phys.

[55] Fairbanks M S, McCarthy D N, Scott S A, Brown S A, Taylor R P (2011). Nanotechnology.

[56] Losa G A, Peretti V, Ciotola F, Cocchia N, De Vico G, Losa G A, Merlini D, Nonnenmacher T F (2005). The Use of Fractal Analysis for the Quantification of Oocyte Cytoplasm Morphology. Fractals in Biology and Medicine.

[57] Gracheva I E, Moshnikov V A, Karpova S S, Maraeva E V (2011). J Phys: Conf Ser.

[58] Cai Y, Zhang Newby B-m (2008). J Am Chem Soc.

[59] Maillard M, Motte L, Ngo A T, Pileni M P (2000). J Phys Chem B.

[60] Deegan R D, Bakajin O, Dupont T F, Huber G, Nagel S R, Witten T A (1997). Nature.

[61] Shen L, Denner F, Morgan N, van Wachem B, Dini D (2020). Sci Adv.

[62] Zhao W, Li Y, Zhang M, Chen J, Xie L, Shi Q, Zhu X (2016). Chem Eng J.

[63] Yin X-T, Zhou W-D, Li J, Wang Q, Wu F-Y, Dastan D, Wang D, Garmestani H, Wang X-M, Ţălu Ş (2019). J Alloys Compd.

[64] Parambhath V B, Nagar R, Ramaprabhu S (2012). Langmuir.

[65] Im J, Shin H, Jang H, Kim H, Choi M (2014). Nat Commun.

[66] Mohamed S H (2012). J Alloys Compd.

[67] Zhang Y, Li D, Qin L, Zhao P, Liu F, Chuai X, Sun P, Liang X, Gao Y, Sun Y (2018). Sens Actuators, B.

[68] Kante I, Devers T, Harba R, Andreazza-Vignolle C, Andreazza P (2005). Microelectron J.

[69] Moshnikov V A, Gracheva I E, An’chkov M G (2011). Glass Phys Chem.

[70] Grachova I E, Maksimov A I, Moshnikov V A (2009). J Surf Invest: X-Ray, Synchrotron Neutron Tech.

[71] Phadungdhitidhada S, Thanasanvorakun S, Mangkorntong P, Choopun S, Mangkorntong N, Wongratanaphisan D (2011). Curr Appl Phys.

[72] Jeun J-H, Kim D-H, Hong S-H (2013). Mater Lett.

[73] Sabri Y M, Kandjani A E, Rashid S S A A H, Harrison C J, Ippolito S J, Bhargava S K (2018). Sens Actuators, B.

[74] Bailly G, Rossignol J, de Fonseca B, Pribetich P, Stuerga D (2015). Procedia Eng.

[75] Fan F, Feng Y, Tang P, Chen A, Luo R, Li D (2014). Ind Eng Chem Res.

[76] Liu X, Zhang J, Yang T, Wang L, Kang Y, Wang S, Wu S (2012). Powder Technol.

[77] Zhang N, Yu K, Li Q, Zhu Z Q, Wan Q (2008). J Appl Phys.

[78] Simon I, Savitsky A, Mülhaupt R, Pankov V, Janiak C (2021). Beilstein J Nanotechnol.

[79] Qin Y, Wang Z, Liu D, Wang K (2017). Mater Lett.

[80] Xiao B, Wang D, Wang F, Zhao Q, Zhai C, Zhang M (2017). Ceram Int.

[81] Zhao Y, Xie Y, Zhu X, Yan S, Wang S (2008). Chem – Eur J.

[82] Bai S, Sun L, Sun J, Han J, Zhang K, Li Q, Luo R, Li D, Chen A (2021). J Colloid Interface Sci.

[83] Guo W, Ma J, Pang G, Wei C, Zheng W (2014). J Mater Chem A.

[84] Pang H, Gao F, Chen Q, Liu R, Lu Q (2012). Dalton Trans.

[85] Wang J, Wei L, Zhang L, Zhang J, Wei H, Jiang C, Zhang Y (2012). J Mater Chem.

[86] Zhao Q, Chuai M, Xiao B, Yang T, Luo Y, Zhang M (2015). New J Chem.

[87] Ji H, Zeng W, Li Y (2019). Nanoscale.

[88] Wang M, Hou T, Shen Z, Zhao X, Ji H (2019). Sens Actuators, B.

[89] Sakai G, Matsunaga N, Shimanoe K, Yamazoe N (2001). Sens Actuators, B.

[90] Yamazoe N, Fuchigami J, Kishikawa M, Seiyama T (1979). Surf Sci.

[91] Deng Y (2019). Semiconducting Metal Oxides for Gas Sensing.

[92] Seal S, Shukla S (2002). JOM.

