# Supplementary material for: Morphology-driven gas sensing by fabricated fractals: A review
Source: Beilstein J Nanotechnol. 2021 Nov 9;12:1187–208. doi: 10.3762/bjnano.12.88 (PMC8593696; doi:10.3762/bjnano.12.88)
Supplement: File 1 — Summary of the fractal structures and their applications. [file Beilstein_J_Nanotechnol-12-1187-s001.pdf]

BEILSTEIN JOURNAL OF NANOTECHNOLOGY

Supporting Information

for

Morphology-driven gas sensing by fabricated fractals: A review

Vishal Kamathe and Rupali Nagar

Beilstein J. Nanotechnol. **2021,** *12,* 1187–1208. doi:10.3762/bjnano.12.88

Summary of the fractal structures and their applications

License and Terms: This is a supporting information file under the terms of the Creative Commons Attribution License (https://creativecommons.org/licenses/by/4.0). Please note that the reuse, redistribution and reproduction in particular requires that the author(s) and source are credited and that individual graphics may be subject to special legal provisions.
The license is subject to the *Beilstein Journal of Nanotechnology* terms and conditions: (https://www.beilstein-journals.org/bjnano/terms)

The Table S1 below summarizes the different fractal structures used for gas sensing applications.

Table S1: Details of fractals used for sensing various gases under specific conditions.

Shape of Fractal	***Size or fractal dimension***	***Material***	***Synthesis Technique***	***Gas sensed***	***Characteristics***	***Type of Sensor***	***Reference***
Fine dendrite-like nanocrystals	1.896 at 300 °C<br>1.884 at 350 °C<br>1.865 at 400 °C<br>1.818 at 450 °C	SnO_2	Sol–gel method & pulsed laser deposition (PLD) technique	Carbon monoxide (CO)	Conc. Range 25-500 ppm, the sensitivity increases with increasing CO conc. and decreasing fractal dimension	Thin Film	[1]
Net-like nanocomposite	Particle size 10-1000 nm	SiO_2-SnO_2 ZnO-SiO_2	Sol–gel method	Acetone and ethanol vapors	Conc. 1000 ppm, Sensitivity to gas-reagents is S > 20	Thin film	[2]
Fractal-like network structure	------	TiO_2	Chemical vapor deposition (CVD) technique	Acetone sensing	Conc. Range 1.2 and 12.5 ppm, Response time 12 s and recovery time 174 s, High repeatability (>98%) in UV-illuminated condition	Conductometric device with finger electrodes	[3]
Fractal	1.8	TiO_2	A flame spray pyrolysis set up used for the TiO_2 fractal formation. Gold plasmonic metasurfaces were obtained through a hole-mask colloidal lithography technique	Ethanol, acetone, and toluene	Localized surface plasmon resonance shifts against the temperature leads to an overall exponential decay trend with decay rates depending on the analyte $\tau_{ethanol}$ = 26 ± 3 °C $\tau_{acetone}$ = 7.4 ± 0.3 °C and $\tau_{toluene}$ = 12 ± 3 °C	Film	[4]

3D hierarchical flowerlike ZnO superstructures (highly dendritic and loose structure)	-----	ZnO	Hydrothermal method	Ethanol and methanol	The flowerlike ZnO is more sensitive than the ZnO microparticles for detecting ethanol and methanol. It showed dynamic response–recovery curve. It can be seen that the sensor response amplitude of flowerlike ZnO to all gas concentrations are much higher than those of ZnO microparticles.	Thin Film	[5]
Nanodendrites and nanowires	The lengths of The nano dendrites are in the range 72–97 nm, which are the same as the diameters of the nanowires. The lengths of the nanowires are in the range of 3–9 µm.	SnO_2	Evaporation–condensation method with VLS growth technique	Nitrogen dioxide (NO_2)	The highest sensor sensitivities for 10, 15, 25 and 50 ppm of NO_2 conc. are 40, 56, 83 and 121, respectively & response time at optimum temp. at 200°C, to be 39, 27, 15 and 6 s for same conc.	Thin Film	[6]
Christmas-tree-like structure	The trunks have lengths in the range 6–10 µm with dia. varying from 190 nm to 200 nm, and the branches have lengths in the range 1–3 µm with dia. varying from 150 nm to 180 nm.	Zn doped NiO	An electrolytic approach combined with subsequent high temperature oxidation	Ammonia (NH_3)	The sensor with doped NiO dendritic crystals gave 5–8 times faster responses and 30–50 times faster recovery speeds than the sensor with pristine NiO dendritic crystals	Interdigitated electrode	[7]

Dendrites	The multilevel branches are composed of well-oriented nanorods with diameters ranging from 60 to 800 nm	ZnO	Cu catalytic Vapor-Liquid-Solid -VLS-growth process	Hydrogen sulfide (H_2S)	Response for 500 ppm of H_2S is 26.4 at 30 °C temperature. The response time and recovery time are about 15–20 and 30–50 s, respectively. It also showed higher selectivity towards other gases.	Two ends of the single dendrite were fixed by conductive silver paste on the oxidized silicon substrate	[8]
Dendrites	The length of leaf-like SnO_2 was about 11-15 µm	SnO_2	Facile template-free hydrothermal synthesis method	Nitrogen dioxide (NO_2)	Leaf-like SnO_2 materials exhibits an excellent selectivity to NO_2 against the other tested gases. The response time and recovery time to 500 ppb NO_2 are resp. about 10 and 46 min. The sensor exhibited good stability during the test for 30 days.	Alumina tube	[9]
Dendrite-like hierarchical structure	The SiNWs with a length of about 35 µm & WO_3 nanowires as branches with a length of about 0.6-1 µm	Composite array of SiNWs and WO_3 NWs	Metal-assisted chemical etching (MACE), through thermal annealing of pre-deposited W film (RF magnetron sputtering technique)	Nitrogen dioxide (NO_2)	Fast response and recovery, especially transient response with response time less than 1s are achieved upon 0.5-5 ppm NO_2 exposure at room temperature.	Silicon substrate	[10]

Mixed morphology of nanowires and nanodendrites	The NWs with the dia. of 50-150 nm and the length of a few 10 µm. The nanodendrites were about 100-300 nm in diameter.	SnO_2 and carbon powder	Carbothermal reduction of SnO_2 in closed crucible (Horizontal alumina tubular furnace was used)	Ethanol	The sensor response was 120 for 1000 ppm of ethanol conc. at 360°C. Response time was in the range of 1-10 s. Recovery time was in the range of 10- several hundred s.	Gold-coated alumina Substrate (Film)	[11]
Hyperbranched Structure	500 nm –1µm	Monoclinic bismuth vanadate	Hydrothermal Treatment	Ethanol and formaldehyde	Higher sensitive to ethanol than formaldehyde at room temperature	-----	[12]
Pine dendritic	400 nm–2 µm	$BiVO_4$ / rGO	Hydrothermal Treatment	Triethylamine	Response = 5.9, response time = 11.4 s, recovery time = 67.2 s at 180°C with excellent selectivity and long-term stability.	-----	[13]

References

1. Chen, Z.; Pan, D.; Zhao, B.; Ding, G.; Jiao, Z.; Wu, M.; Shek, C.-H.; Wu, L. C. M.; Lai, J. K. L. *ACS Nano* **2010,** *4,* 1202–1208. doi:10.1021/nn901635f
2. Gracheva, I. E.; Moshnikov, V. A.; Karpova, S. S.; Maraeva, E. V. J. Phys.: Conf. Ser. 2011, 291, 012017. doi:10.1088/1742-6596/291/1/012017
3. Sabri, Y. M.; Kandjani, A. E.; Rashid, S. S. A. A. H.; Harrison, C. J.; Ippolito, S. J.; Bhargava, S. K. Sens. Actuators, B 2018, 275, 215–222. doi:10.1016/j.snb.2018.08.059
4. Fusco, Z.; Rahmani, M.; Bo, R.; Verre, R.; Motta, N.; Käll, M.; Neshev, D.; Tricoli, A. Adv. Mater. (Weinheim, Ger.) 2018, 30, 1800931. doi:10.1002/adma.201800931
5. Liu, X.; Zhang, J.; Yang, T.; Wang, L.; Kang, Y.; Wang, S.; Wu, S. Powder Technol. 2012, 217, 238–244. doi:10.1016/j.powtec.2011.10.032
6. Mohamed, S. H. J. Alloys Compd. 2012, 510, 119–124. doi:10.1016/j.jallcom.2011.09.006

7. Wang, J.; Wei, L.; Zhang, L.; Zhang, J.; Wei, H.; Jiang, C.; Zhang, Y. J. Mater. Chem. 2012, 22, 20038–20047. doi:10.1039/c2jm34192a
8. Zhang, N.; Yu, K.; Li, Q.; Zhu, Z. Q.; Wan, Q. J. Appl. Phys. 2008, 103, 104305. doi:10.1063/1.2924430
9. Zhang, Y.; Li, D.; Qin, L.; Zhao, P.; Liu, F.; Chuai, X.; Sun, P.; Liang, X.; Gao, Y.; Sun, Y.; Lu, G. Sens. Actuators, B 2018, 255, 2944–2951. doi:10.1016/j.snb.2017.09.115
10. Qin, Y.; Wang, Z.; Liu, D.; Wang, K. Mater. Lett. 2017, 207, 29–32. doi:10.1016/j.matlet.2017.07.042
11. Phadungdhitidhada, S.; Thanasanvorakun, S.; Mangkorntong, P.; Choopun, S.; Mangkorntong, N.; Wongratanaphisan, D. Curr. Appl. Phys. 2011, 11, 1368–1373. doi:10.1016/j.cap.2011.04.007
12. Zhao, Y.; Xie, Y.; Zhu, X.; Yan, S.; Wang, S. Chem. – Eur. J. 2008, 14, 1601–1606. doi:10.1002/chem.200701053
13. Bai, S.; Sun, L.; Sun, J.; Han, J.; Zhang, K.; Li, Q.; Luo, R.; Li, D.; Chen, A. J. Colloid Interface Sci. 2021, 587, 183–191. doi:10.1016/j.jcis.2020.10.113
